# Characteristics and comorbidities of patients on opioid agonist therapy in Switzerland: A descriptive analysis of the nationwide SAMMSU cohort

**DOI:** 10.1016/j.dadr.2025.100404

**Published:** 2025-12-16

**Authors:** Michael Lütolf, Andrea Bregenzer, Philip Bruggmann, Alberto Moriggia, Claude Scheidegger, Katharina Hensel-Koch, Erika Castro Batänjer, Maria Christine Thurnheer, Pascale Della Santa, Oliver Senn, Thomas Grischott

**Affiliations:** a Institute of Primary Care, University Hospital Zurich, University of Zurich, Zurich, Switzerland; b Department of Infectious Diseases and Infection Prevention, Cantonal Hospital Aarau, Aarau, Switzerland; c Arud Centre for Addiction Medicine, Zurich, Switzerland; d Epatocentro Ticino Foundation, Lugano & Ingrado Addiction Services, Lugano, Switzerland; e Independent, Basel & Swiss Hepatitis, Zurich, Switzerland; f Addiction Support Foundation, St. Gallen, Switzerland; g Private Practice, Lausanne & HepC Virtual Clinic, Lausanne, Switzerland; h Department of Infectious Diseases, University Hospital Bern, University of Bern, Bern, Switzerland; i Les Toises, Sion, Switzerland

**Keywords:** Opioid use disorder, Opioid agonist treatment, Cohort description, Epidemiology, Switzerland

## Abstract

**Background:**

Opioid agonist therapy (OAT) is the gold standard of treatment for opioid dependence and a cornerstone of Swiss drug policy. The Swiss Association for the Medical Management in Substance Users (SAMMSU) cohort was established to monitor health trends and improve care for OAT patients across Switzerland.

**Methods:**

Baseline and follow-up data collected from eight centres between 2014 and 2024 were analysed descriptively, including demographic and psychosocial characteristics, substance use history, prescribed OAT, co-medications, and somatic and psychiatric comorbidities.

**Results:**

During the study, the SAMMSU cohort included 1 502 participants. Median individual age at registration was 44.3 years, rising to a cohort median of 50.9 years by the end of 2024; 75.7 % of participants were male. Lifetime heroin use was reported by 97.2 %, with 73.2 % having a history of intravenous drug use. Ongoing illicit and intravenous drug use declined over time, while prescribed OAT shifted from methadone to long-acting morphine and diacetylmorphine. The most prevalent lifetime somatic comorbidities were hepatitis C (56.5 %), (pre)hypertension (18.6 %), musculoskeletal disorders (13.8 %), and needle abscesses (13.7 %). Psychiatric disorders – primarily affective (34.8 %), personality (23.2 %), and anxiety disorders (18.0 %) – contributed to multimorbidity and a high prevalence of polypharmacy (49.2 %). There were 120 deaths, mainly from malignancy, overdose, and liver failure, with a median age at death of 51.6 years.

**Conclusion:**

SAMMSU cohort trends corroborate the effectiveness of OAT in reducing illicit drug use and underscore the need for OAT services to evolve from an addiction-focused model to comprehensive chronic care for an ageing and highly vulnerable population.

## Highlights

• The ageing OAT cohort increasingly shows a chronic multimorbidity profile.
• Hypertension, diabetes, COPD, and osteoporosis appear at markedly younger age.
• Mood disorders and schizophrenia drive the cohort’s high psychiatric comorbidity.
• Over half of OAT patients exhibit polypharmacy, often including psychotropic drugs.
• OAT services must evolve from addiction-focused to comprehensive chronic care.

## 1 Introduction

### 1.1 Opioid agonist therapy in Europe and Switzerland

Opioid dependence is a serious chronic relapsing health condition (World Health Organization, 2009) affecting an estimated 40.5 million people worldwide (Degenhardt et al., 2019). Opioid agonist therapy (OAT) is a treatment for this dependence syndrome that involves the long-term prescription of opioid agonist medications (Pompidou Group, 2017), such as methadone, buprenorphine, sustained-release morphine, or diacetylmorphine, and is complemented by supportive measures, including somatic and psychiatric care as well as social work services (Bundesamt für Gesundheit, 2013).

Numerous studies have demonstrated the effectiveness of OAT in improving health and social outcomes of people with opioid dependence. This includes reduced illicit opioid use along with a decrease in intravenous drug use and its associated risks; significantly lower all-cause, overdose and suicide mortality; reduced criminal activity; increased HIV and HCV testing and treatment; and improved employment rates and quality of life compared to no treatment or alternative treatment forms (Degenhardt et al., 2019, Hubbard et al., 2003, Pompidou Group, 2017, Santo et al., 2021). Nevertheless, access to OAT remains uneven globally, with fewer than 10 % of individuals in need of treatment actually receiving it (World Health Organization, 2023). In the European Union, about half of the high-risk opioid users received OAT in 2022, amounting to an estimated 513 000 recipients. Treatment coverage varies greatly between countries, ranging from around 10 % in Latvia to 80–100 % in Spain (European Union Drugs Agency, 2025).

Switzerland experienced a peak in heroin consumption during the 1980s and early 1990s, characterized by open drug scenes that attracted users from across the country and even neighbouring countries. These scenes contributed to an exceptionally severe HIV epidemic by international standards (Uchtenhagen, 2010) and to high rates of overdose mortality. In response, Switzerland developed a comprehensive four-pillar policy – founded on prevention, treatment, harm reduction, and law enforcement – to address the heroin epidemic and visible drug problems. OAT plays a crucial role in both the treatment and harm reduction pillars. Today, Swiss drug policy is widely regarded as a success story. It has halved overdose-related deaths, reduced the number of new heroin users, and significantly lowered HIV infections and drug-related crime. Additionally, it has led to the near-complete disappearance of drug-related problems from public visibility (Bundesamt für Gesundheit, 2013, Pollien, 2023, Uchtenhagen, 2010).

In Switzerland, methadone has been approved as OAT medication covered by compulsory health insurance since the 1970s, diacetylmorphine since the 1990s, buprenorphine since the early 2000s, and extended-release morphine approximately a decade later. Mandatory reporting of the specific OAT is required, and the prescription of diacetylmorphine is restricted to specialized institutions within specific cantons and additionally requires authorization from federal authorities. It has been estimated that roughly 80 % of drug users receive care within an OAT program (Bregenzer et al., 2022), a figure that ranks high in international comparisons. In absolute terms, approximately 17 800 individuals received OAT in 2022, according to Swiss national statistics, a figure that has shown a slight decline in recent years. About half of OAT prescriptions are issued by physicians in private practice, while the remainder are prescribed by physicians working in specialised institutions or prisons (Gmel and Labhart, 2023, Labhart and Amos, 2024). Actual dispensing of the OAT substances takes place predominantly in pharmacies (53 % of cases), but also in specialized institutions (33 %) or in private practices by the prescribing physicians themselves (13 %) (Nationale OAT-Statistik, 2023). Directly observed treatment is applied on an individual basis, primarily for patients at risk of losing or misusing their take-home doses.

Improved treatment has significantly increased the life expectancy of opioid users and thus led to an ageing OAT population. The median age increased from 40 to 48 years between 2010 and 2020, resulting in higher prevalence rates of chronic diseases, neurocognitive impairments, and disability (Schwarz et al., 2024). As OAT patients age and direct drug-related harm decreases, their risks shift to somatic comorbidities and mortality, with chronic diseases emerging earlier and at higher rates than in the general population (Bech et al., 2019, Medved et al., 2020).

### 1.2 Scope and data coverage of the SAMMSU cohort

This demographic shift, along with the high burden of early-onset comorbidities, underscores the need for comprehensive data to inform and refine treatment strategies in this population, both in relation to OAT itself and the growing imperative of chronic disease management.

The SAMMSU cohort, established by the Swiss Association for the Medical Management in Substance Users (SAMMSU), aims to improve medical management for substance users in Switzerland. It includes patients from eight participating centres (Aarau, Zurich, Lugano, Basel, St. Gallen, Lausanne, Geneva, and Bern), representing approximately 8 % of the estimated total OAT population in Switzerland. Lack of scientific study experience in several centres, lack of resources in the experienced ones, and independent physicians not contributing to the cohort pose obstacles to recruitment and have so far prevented higher representativeness. The SAMMSU cohort has collected longitudinal socio-demographic and medical data – including data on drug use, risk behaviour, comorbidities, medications, and laboratory results – on a regular basis since its inception in 2014 (SAMMSU, 2024).

### 1.3 Study aim

The present study aims to provide a descriptive analysis of the SAMMSU cohort to elucidate cohort characteristics, patients’ demographic profiles, substance use patterns, and prescribed OAT medications. Furthermore, it analyses their comorbidities and concomitant medications. The data generated are intended to enhance understanding of the evolving needs of patients receiving OAT, inform further scientific research, and support the development of improved treatment strategies.

## 2 Methods

### 2.1 Cohort design, inclusion criteria, and ethics approval

The SAMMSU cohort is an ongoing, open cohort without a pre-specified endpoint other than participant death (SAMMSU, 2024). Patients over 18 years of age enrolled in an OAT programme for at least one day at one of the participating centres are eligible for inclusion upon providing written informed consent. In principle, all eligible patients should be invited to join the cohort; however, actual recruitment is carried out in Aarau and Zurich by research staff but in the other centres at the discretion of the clinicians. The SAMMSU cohort protocol was approved by the competent ethics committees of all participating centres, led by Ethikkommission Ostschweiz (EKOS) (BASEC No. PB_2016_01545).

### 2.2 Data collection

At registration, a baseline set of socio-demographic and medical data – including information on drug use, risk behaviour, comorbidities, medications, vaccinations and laboratory results – is collected from the patients’ medical records and supplemented with data from a brief interview on risk behaviour and living conditions. The list of variables collected is available in Appendix G. Subsequently, longitudinal follow-up data are collected periodically (annually, as per protocol). Follow-ups are tracked using lists organized by follow-up due dates, prioritizing patients with the earliest due dates. Often, follow-ups can be completed through review of medical records collected since the previous follow-up. Relevant information collected during regular patient appointments throughout the observation period is also integrated into the dataset.

### 2.3 Data management, security, and access

Data are entered into the database via an online data entry system using single data entry. No directly identifying personal information (e.g., name or address) is entered; instead, a 5-digit patient ID is used and each centre keeps a code list. The central database is web-based and was constructed using SecuTrial® (interActive Systems GmbH, Berlin, Germany). It is maintained by the informatics division of the Clinical Trial Unit at the University of Basel. The complete data set is available for analyses within specific research projects, and project proposals can be submitted via the SAMMSU website (SAMMSU, 2024).

### 2.4 Data analysis

Descriptive analyses (location and dispersion measures, proportions, relative risks, Yule’s Q, etc.) were performed using R (version 4.4.1, R et al., 2024) and using the tidyverse (Wickham et al., 2019) and common standard packages.

The dataset used for analysis comprised all data collected from the initiation of the cohort until the end of 2024. In all analyses, percentages were calculated with denominators that excluded missing values. (Where applicable, and if not reported directly alongside the relevant percentages, counts of available or missing values are provided in the Appendix.) The two Geneva-based centres (Hôpitaux Universitaires and Fondation Phénix) were combined and treated as a single centre.

Baseline analyses were conducted using data from the initial registration visit. Longitudinal analyses were carried out either by individual patient follow-ups (i.e., registration, follow-up 1, follow-up 2, etc.) or by calendar year, which additionally captures general cohort trends influenced by new participant registrations and study discontinuations, rather than just changes within individual participants.

When comparing baseline information from the registration visit with follow-up data, the latest available follow-up information for each participant was used. For participants with no recorded follow-ups or no additional data entry in the relevant categories, baseline information served as fallback.

Where relevant information was found in free-text responses, particularly regarding reasons for study discontinuation, the entries were assigned to predefined categories, or new categories were created where necessary.

## 3 Results

### 3.1 Cohort description

**Fig. 1:**
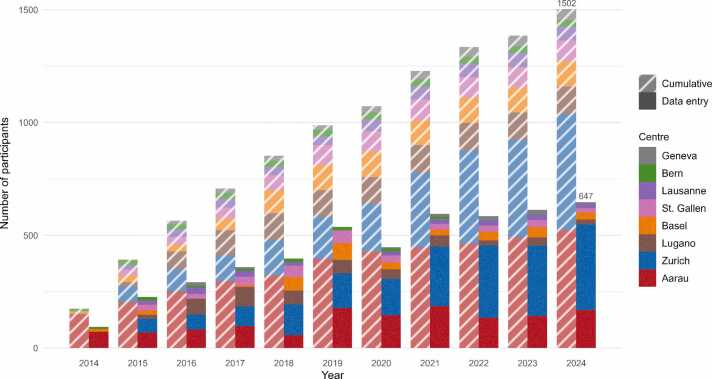
Evolution of the SAMMSU cohort 2014–2024. Number of cumulatively enrolled participants (hatched) and of participants with data entry in specific years (solid), by centre.

A total of *N* = 1 502 participants were registered in the SAMMSU cohort across the eight centres by the end of 2024. The number of cumulatively enrolled participants over the years is shown as hatched bars in Fig. 1. The highest numbers of inclusions were seen between 2015 and 2021, except for 2020, likely due to the impact of COVID-19. Mean follow-up duration (from registration to documented cohort exit or the end of 2024, whichever came first, and excluding 11 participants with obviously erroneous follow-up dates and a further 44 participants lacking both a documented exit and any follow-up for five years or longer) is 4.7 years. In addition to the registration visit, participants had between 0 and 9 follow-ups, with an average of 2.3 follow-ups (3.2 among the 72.8 % of participants with at least one follow-up). Detailed data, stratified by centre, on cumulative numbers of included participants, follow-ups per patient, and time spent in the cohort are provided in Table A.1 in Appendix A, and Figure A.1 (ibid.) shows proportions of participants per number of follow-ups.

The numbers of participants with data entry (beyond consent and basic demographics) in any given year are shown as solid bars in Fig. 1. On average, 47 % of all participants included up to a given year had at least one data entry in that year. For example, of the 1 502 participants cumulatively registered by 2024, 647 had a data entry in 2024.

A total of 590 participants exited the cohort, including 120 deaths and 460 non-death exits (10 unknown). The crude all-cause mortality rate within the cohort was approximately 16.3 deaths per 1 000 person-years (95 % CI 13.6–19.3). The most common reasons for non-death exits were loss to follow-up, transfer of care to a non-cohort physician, and patient wish. Detailed information on cohort exit reasons can be found in Table A.2 in Appendix A, and Table A.3 (ibid.) lists places of death.

### 3.2 Patient characteristics

Baseline cohort demographics and social variables are summarised in Table 1. At registration, the median age was 44.3 years; the cohort comprised 75.7 % male and 97.6 % white participants (see Figure B.1 in Appendix B for age and gender distribution). Approximately half (49.8 %) had normal weight, whereas 43.9 % were overweight or obese, and 6.3 % were underweight. In the six months preceding baseline, 59.0 % of participants were unemployed, and 78.0 % received financial support (pensions, social services, unemployment insurance, invalidity insurance, or other sources). Of all participants, 17.3 % lived with assistance (including housing/domestic or medical support), while 13.0 % resided in care homes. Only small minorities of participants had been imprisoned (5.6 %) or homeless (3.6 %) for at least three of the past six months.

**Table 1:** Basic patient demographics and social variables at registration. Social variables were recorded for the six-month period before the baseline interview.

		Total *n*		At registration
Age	years	1502	median (IQR)	44.3 (36.1–50.6)
Gender	- male	1501	# (%)	1137 (75.7 %)
	- female		# (%)	364 (24.3 %)
Ethnicity	- white	1500	# (%)	1464 (97.6 %)
	- Asian		# (%)	18 (1.2 %)
	- Hispano-American		# (%)	10 (0.7 %)
	- black		# (%)	5 (0.3 %)
	- other		# (%)	3 (0.2 %)
BMI category	- underweight (<18.5 kg/m^2^)	1434	# (%)	91 (6.3 %)
	- normal (18.5–24.9 kg/m^2^)		# (%)	714 (49.8 %)
	- overweight (25.0–29.9 kg/m2)		# (%)	408 (28.5 %)
	- obese (≥30.0 kg/m^2^)		# (%)	221 (15.4 %)
Unemployed		1384	# (%)	816 (59.0 %)
Receiving financial support		1310	# (%)	1022 (78.0 %)
Been imprisoned (for ≥3 months)		1394	# (%)	78 (5.6 %)
Been homeless (for ≥3 months)		1405	# (%)	50 (3.6 %)
Living with assistance		1393	# (%)	241 (17.3 %)
Living in care home		239	# (%)	31 (13.0 %)
In stable partnership		1369	# (%)	572 (41.8 %)
Commercial sex work		953	# (%)	15 (1.6 %)

After excluding cohort exits (i.e., deaths and drop-outs), the median age of the remaining participants at the end of 2024 was 50.9 years. Comparison of the latest available data from these participants with baseline data from all participants revealed slight decreases in the proportions of those receiving financial support (from 78.0 % to 74.9 %), experiencing homelessness (from 3.6 % to 3.0 %), or requiring assisted living (from 17.3 % to 13.2 %). In contrast, the proportion of participants residing in care homes increased slightly, from 13.0 % to 14.9 %). The percentage of participants engaged in commercial sex work decreased from 1.6 % to 0.9 %. While the median age of the patients followed in the cohort increased over time, the median age at registration decreased slightly, from 45.8 years in the first five years (2014–2018) to 43.0 years in the last five years (2020–2024). Additional follow-up demographics are presented in Table B.1, with Figure B.2 (both in Appendix B) zooming in on the evolution of age at registration over time.

### 3.3 Substance use

At registration, lifetime heroin use was reported by 97.2 % of participants, with a median year of first use in 1993 and a median age at first use of 19.4 years. Cocaine use was reported by 92.3 %. Lifetime use of cannabis was similarly high at 89.8 % but typically began at an earlier age (median year of first use: 1987; median age: 15.5 years). Benzodiazepine use was less common, reported by 68.6 %. Detailed numerical data on lifetime substance use can be found in Table C.1 in Appendix C, and the distributions of year of first use and age at first use for each substance are shown in Figure C.1 (ibid.).

Table C.1 also shows that nicotine use was nearly universal (97.1 %) and typically started early (median initiation age: 14.7 years). At registration, nicotine users reported a median number of 29 pack-years. In contrast, e-cigarette use was uncommon (13.2 %) and typically began much later (median year: 2020; median age: 37.5 years). At registration, 73.2 % of participants reported having ever used drugs intravenously, while intranasal use was even more prevalent at 90.0 %.

**Fig. 2:**
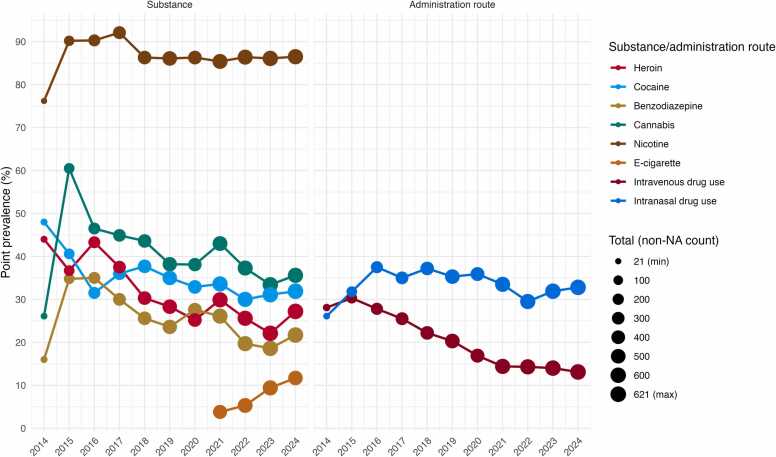
Substance use patterns (left) and routes of administration (right) observed in the cohort over time. NA: missing value.

Also taking follow-up data into account, ongoing use of most individual substances declined over the study period, as illustrated in Fig. 2: heroin use decreased from 41.3 % in the first three years (2014–2016) to 25.0 % in the last three years (2022–2024), cocaine from 40.0 % to 31.0 %, cannabis from 44.4 % to 35.4 %, and benzodiazepine use from 28.6 % to 20.0 %. In contrast, nicotine use remained high (85.6–86.3 %) despite an increasing use of e-cigarettes towards the end of the study period. Ongoing intravenous drug use in the cohort declined substantially over the same period, with the average yearly prevalence dropping from 28.7 % in the first three years to 13.8 % in the last three years.

**Fig. 3:**
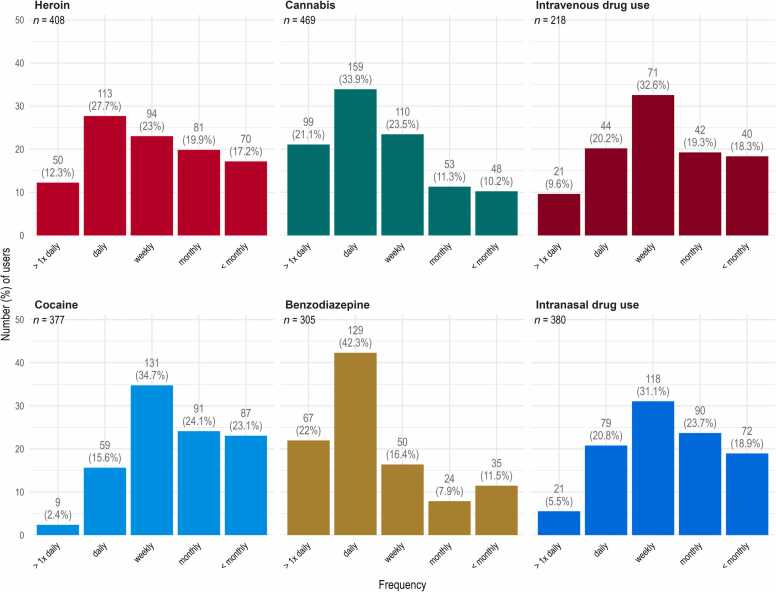
Frequency of substance use at registration, by substance or administration route.

Fig. 3 illustrates the frequency of use for the different substances and corresponding routes of administration. The majority of cocaine users (82.0 %) reported consuming the substance weekly or less frequently, whereas among benzodiazepine users, most (64.3 %) reported daily or multiple daily use.

Additional figures illustrating cigarette pack-year distribution, as well as daily alcohol consumption are provided in Appendix C (Figures C.2-C.3).

### 3.4 OAT and other substitution medication

At registration, 95.5 % of the *N* = 1 502 cohort participants were receiving OAT, 2.2 % were not currently under OAT due to treatment interruption or cessation attempts, and for another 2.2 %, the information was missing. Of those receiving OAT at registration, 5.5 % were no longer on OAT at their last follow-up (where this information was available), and 29.1 % of these subsequently dropped out of the cohort. Conversely, among the 2.2 % not receiving OAT at registration, 17.6 % restarted treatment during the study period.

**Fig. 4:**
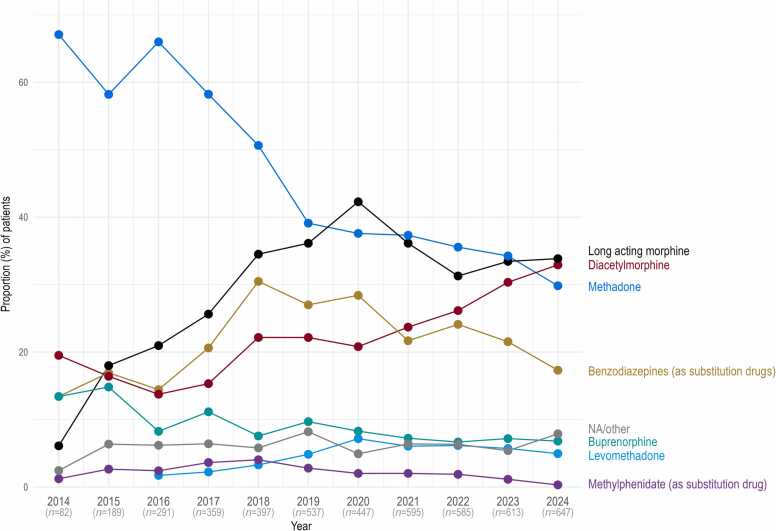
Proportions of patients receiving specific OAT or other substitution medications, by year.

Fig. 4 presents the percentage of participants receiving specific OAT or other substitution medications over time. Between 2014 and 2024, the prevalence of methadone prescription in the cohort decreased from 67.1 % to 29.8 %. In contrast, the use of long-acting oral morphine (morphine sulphate) increased from 6.1 % to 33.8 %, and diacetylmorphine prescription prevalence rose from 19.5 % to 32.9 %. Further details on subgroups of long-acting morphine and benzodiazepines are provided in Figure D.1 in Appendix D.

OAT was prescribed by a medical institution specializing in addiction care for 78.6 % of participants, by a general practitioner for 19.5 %, and by a psychiatrist for 1.9 %. The place of dispensation was a specialized institution (including ambulatory services) for 78.7 % of participants, a pharmacy for 20.3 %, and a medical practice for 1.0 % (Figure D.2 in Appendix D).

### 3.5 Comorbidity

At registration, the somatic conditions with the highest recorded lifetime prevalence were HCV infections (56.5 %), major surgery (23.4 %; most commonly surgeries of the extremities and abdomen), arterial (pre)hypertension (18.6 %), musculoskeletal disorders (13.8 %), needle abscesses requiring surgery (13.7 %), epilepsy (11.7 %; mostly attributable to substance withdrawal or intoxication), COPD (9.9 %), HIV infection (9.2 %), malignancies (8.4 %; most frequently of the breast, uterus, meninges, anal canal, or liver), thrombosis (7.5 %), hypogonadism (6.3 %), and cardiovascular disease (6.2 %; most commonly chronic ischaemic heart disease and pulmonary heart disease).

Psychiatric comorbidities were also highly prevalent. Those most frequently recorded at registration were affective disorders (34.8 %), personality disorders (23.2 %), anxiety disorders (18.0 %), and schizophrenia (17.5 %). Furthermore, 17.2 % of participants had a history of suicide attempt(s). Comprehensive prevalence data, including data from follow-ups, are provided in Appendix E (Table E.1 and E.2). Figure E.1 (ibid.) presents patient counts for the five most prevalent somatic conditions and the five most prevalent psychiatric comorbidities, as well as their respective overlaps.

Substance use patterns were associated with specific diagnoses. Most notably, intravenous drug use was strongly associated with needle abscesses (risk ratio 31.5; for confidence intervals see Table E.3 in Appendix E), HIV (7.2), HCV (6.9), endocarditis (4.2), sexually transmitted diseases (3.6), osteoporosis (3.3), and thrombosis (2.8). Hypogonadism, osteoporosis, pancreatitis, endocarditis, and peripheral artery disease were only observed among (illicit) heroin users. Some diagnoses were also found to be strongly associated, forming clusters. The most prominent cluster comprised endocarditis, thrombosis, peripheral artery disease, needle abscesses, and cardiovascular disease (pairwise odds ratios ranging from 2.2 for needle abscess/cardiovascular disease to 13.6 for peripheral artery disease/thrombosis). Association tables and network visualisations are available in Appendix E (Table E.3, Figures E.2 and E.3).

During the study period, 120 deaths were recorded. The leading causes of death were malignancy (17.8 % of deaths with known causes), narcotics overdose (13.7 %), liver failure or cirrhosis (9.6 %), COPD, pneumonia, and sepsis (each 6.8 %). However, the cause of death remained unknown in 47 cases. Median age at death was 51.6 years. Of all deaths with known places, 46.2 % occurred at home, 35.8 % in hospital acute care units, and 13.2 % in chronic care institutions. For details, see Tables A.2 and A.3 in Appendix A.

### 3.6 Medication

**Fig. 5:**
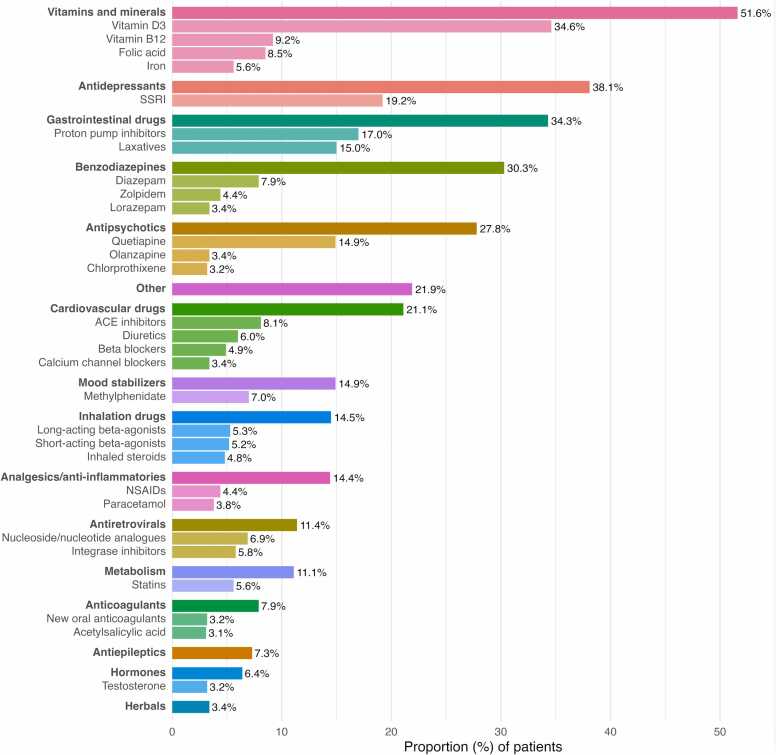
Non-OAT medication. Prevalence of drug classes (bold label, darker color) and subclasses (lighter color) at the end of 2024 (excluding OAT and other substitution medication). Only classes/subclasses with prevalence ≥ 3.0 % are shown.

Excluding OAT and other substitution medication, a total of 10 655 drug prescriptions were (ever) recorded in the cohort, of which 5 448 were ongoing at the end of 2024 (based on last visit data). A total of 1 295 patients had at least one prescription ever, and 1 171 patients had at least one (ongoing) prescription at the end of 2024. Fig. 5 shows the percentage of patients receiving non-OAT medications as of the end of 2024, by drug class and subclass. The most prevalent classes were vitamins and minerals, antidepressants, gastrointestinal drugs, benzodiazepines, antipsychotics, and cardiovascular medications. Complete patient and prescription counts (both ever and ongoing) are provided in Table F.1 in Appendix F.

The average number of ongoing drugs per patient was 3.9 when OAT and other substitution medications were excluded (Figure F.1 in Appendix F), and 5.3 when they were included. Polypharmacy – here defined as the use of at least five concomitant drugs including OAT and other substitution medications – was present in 49.2 % of patients at the end of 2024.

When considering all prescriptions recorded in the cohort rather than just ongoing medication at the end of 2024, drugs against viral hepatitis were also frequent (in 44.9 % of patients, compared to 0.9 % of patients with ongoing use at the end of 2024). All drug classes were more frequently prescribed at the last visit compared to the first, except for hepatitis drugs (Figure F.2 in Appendix F).

## 4 Discussion

### 4.1 Age and multimorbidity

The present study summarizes demographic and clinical characteristics of the SAMMSU cohort. It shows an ageing cohort (median age rising from approximately 44 to almost 51 years within a decade despite the trend towards including younger patients following the initial cohort’s focus on hepatitis C, which primarily affected older patients undergoing OAT) with a high burden of multimorbidity.

This includes diagnoses that have been commonly associated with drug use, like HCV (lifetime prevalence in the cohort: 56.5 %; approximately 80 times higher than the general Swiss population estimate of 0.7 % (Zahnd et al., 2017)), HIV (cohort: 9.2 %; approximately 46 times higher than the general Swiss population estimate of 0.2 % (Bundesamt für Gesundheit, 2024; Kohler et al., 2015)), needle abscesses, epilepsy, thrombosis, and hypogonadism.

As drug dependence stabilizes through OAT and HCV becomes largely curable, the health risks of patients on OAT are increasingly shifting from directly drug-related risks toward the non-communicable chronic diseases prevalent in the general population, for example arterial hypertension (cohort: 14.5 % at baseline; estimated prevalence in the general population of 35–44-year olds: 5.3 %, in 45–54-year-olds: 13.4 % (Bundesamt für Statistik, 2023)), malignancies (cohort: 8.4 %; estimated lifetime risk in the general population at 45 years: 2.9 %, at 60 years: 10.0 % (Six et al., 2017)), cardiovascular disease, osteoporosis (cohort: 5.9 % at baseline, rising to 8.1 % at last follow-up; estimated prevalence in the general population of 45–54-year-olds: 0.9 %, in 65–74-year-olds: 8.3 %), asthma (cohort: 5.5 %; estimated prevalence in the general population: 5.1 %), and diabetes mellitus (cohort: 4.5 %; estimated prevalence in the general population of 35–44-year-olds: 1.9 %, in 45–54-year-olds: 4.9 % (Bundesamt für Statistik, 2023)).

Particular attention should be given to COPD, which is more prevalent in this cohort (9.9 % at baseline, 14.8 % at follow-up) than in any age group in the general population (estimated prevalence overall: 2.4 %, in 35–44-year-olds: 1.4 %, in ≥75-year-olds: 5.6 % (Bundesamt für Statistik, 2023)) and is expected to increase further, given the high proportion of lifetime (>97 %) or continued (>86 %) smokers and their median smoking history of 29 pack-years.

Psychiatric conditions further exacerbate the comorbidity burden, with elevated rates of affective disorders (cohort: 34.8 %; general population lifetime prevalence estimate: 24.2 % (Angst et al., 2005)) and schizophrenia (cohort: 17.5 %; general population point prevalence estimate: 0.4 % (Pletscher et al., 2015)).

Half of the OAT patients meet or exceed the polypharmacy threshold, taking an average of 5.3 medications – compared to under 20 % prevalence of polypharmacy and a median of 2 medications among 41–64-year-olds in the general population (Rachamin et al., 2022). Particularly noteworthy is the higher prevalence of psychotropic medication prescriptions, specifically antidepressants (38.1 % versus an estimated 9.0 % (Amrein et al., 2023)), benzodiazepines and Z-drugs (30.3 % versus 10.5 % (Landolt et al., 2021)), and antipsychotics (27.8 % in the cohort).

In summary, the chronic multimorbidity profile of ageing OAT patients increasingly resembles that of the general ageing population; however, many chronic diseases tend to manifest a decade or more earlier, and OAT patients continue to face additional drug-related health burdens.

### 4.2 OAT effectiveness and shift in prescribed substances

The study supports the well-established effectiveness of OAT in reducing illicit drug use, particularly intravenous use and its associated risks. This is also reflected in the cohort’s comparatively low mortality rate of 16 ‰ per annum, with most reported causes of deaths attributable to somatic comorbidities rather than directly to opioids or crime. Recent meta-analyses reported similar all-cause mortality rates in cohorts approximately ten years younger: 16 ‰ regardless of OAT status (Bahji et al., 2019); 11 ‰ among patients on methadone or buprenorphine OAT and 24 ‰ among those not receiving any OAT (Santo et al., 2021).

A relevant shift in the substances used for OAT is evident: methadone is being partially replaced by long-acting morphine and diacetylmorphine. This development is likely driven largely by new patients entering the cohort, including those previously treated by general practitioners and subsequently referred to specialised centres (which participate in the cohort) for a medication switch. In contrast, changes of the OAT medication within individual patients account for only a smaller portion of this shift.

### 4.3 Limitations

This work is limited to a descriptive characterisation of the cohort data. Inferential statistical testing of specific hypotheses – for example, regarding risk factors, outcomes, or even interventions – is beyond the scope of this analysis and may be explored in future studies.

We did not place a particular focus on HCV and HIV in this work, as these conditions have already been addressed in previous analyses of this (Bregenzer et al., 2021) and other specialised cohorts (Prasad et al., 2007, Scherrer et al., 2022, Weber et al., 2015).

Accurately capturing benzodiazepine use (and, to a lesser degree, methylphenidate use) proved challenging due to their somewhat ambiguous categorisation as either illicit substances, substitution medications, or other prescribed medications, and the ensuing uncertainty as to whether they were categorised consistently across all centres.

Longitudinal data were available for only a subset of participants with baseline data. While almost three-quarters of participants had at least one follow-up, the average number of follow-ups per patient was relatively low at 2.3, and extended follow-up (i.e., five or more follow-ups) was observed in only one-sixth of registered participants. This limitation could introduce bias – for example, participants with more stable health may be overrepresented among those with longer follow-up – and calls for cautious interpretation of findings from longitudinal analyses depending on individual cohort retention.

Limited coverage of the national OAT population, combined with the fact that the majority of cohort participants were recruited through and treated within highly specialized and organized institutional settings, constrains representativeness. Consequently, the cohort likely reflects a better-cared-for but also sicker group than the broader Swiss OAT population and underrepresents patients receiving OAT under the care of general practitioners. After 2020, a smaller number of centres – particularly Zurich and Aarau – accounted for an increasing share of registrations and data entries, potentially driving trends in OAT regimen types and making the dataset less representative of other regions that were more strongly represented initially.

### 4.4 Conclusion

Our findings from the SAMMSU cohort further corroborates the success of OAT in reducing illicit drug use and highlights the importance of continued observation and research within the OAT population. Considering the increasing and often earlier-onset multimorbidity, OAT services must shift from a predominantly addiction-focused model to one capable of managing comprehensive, chronic care for an ageing and highly vulnerable population.

## CRediT authorship contribution statement

**Philip Bruggmann:** Writing – review & editing, Resources, Investigation, Conceptualization. **Alberto Moriggia:** Writing – review & editing, Resources. **Michael Lütolf:** Writing – review & editing, Writing – original draft, Visualization, Methodology, Investigation, Formal analysis, Data curation, Conceptualization. **Andrea Bregenzer:** Writing – review & editing, Resources, Investigation, Conceptualization. **Claude Scheidegger:** Writing – review & editing, Resources. **Katharina Hensel-Koch:** Writing – review & editing, Resources. **Erika Castro Batänjer:** Writing – review & editing, Resources. **Oliver Senn:** Writing – review & editing, Supervision, Resources, Project administration, Investigation, Conceptualization. **Thomas Grischott:** Writing – review & editing, Writing – original draft, Visualization, Validation, Supervision, Project administration, Methodology, Investigation, Formal analysis, Conceptualization. **Maria Christine Thurnheer:** Writing – review & editing, Resources. **Pascale Della Santa:** Writing – review & editing, Resources.

## Funding

This research did not receive any specific grant from funding agencies in the public, commercial, or not-for-profit sectors.

## Declaration of Competing Interest

The authors declare that they have no competing interests.

## Glossary

OAT: Opioid agonist therapy
SAMMSU: Swiss Association for the Medical Management in Substance Users
