# Supplementary material for: Characteristics and comorbidities of patients on opioid agonist therapy in Switzerland: A descriptive analysis of the nationwide SAMMSU cohort
Source: Drug Alcohol Depend Rep. 2025 Dec 16;18:100404. doi: 10.1016/j.dadr.2025.100404 (PMC12807839; doi:10.1016/j.dadr.2025.100404)
Supplement: Supplementary file 1 — Supplementary material [file mmc1.docx]

Appendix A: Cohort characteristics

Table A.1: Cumulative number of registered patients; number of follow-ups per patient (excluding the initial registration visit); and time in cohort (as defined in the main paper) since patient inclusion, in total and per centre.

| Centre | Number of registered patients | Number of follow-ups  per patient, ∅ (sd) | Time spent in the cohort per patient (years), ∅ (sd) |
| --- | --- | --- | --- |
| *Total* | *1502* | *2.3 (2.3)* | *4.7 (3.0)* |
| Aarau | 525 | 1.7 (1.7) | 5.8 (3.0) |
| Zurich | 513 | 2.9 (2.6) | 3.5 (2.7) |
| Lugano | 121 | 3.0 (2.2) | 7.1 (2.2) |
| Basel | 114 | 2.2 (2.3) | 3.8 (2.3) |
| St. Gallen | 90 | 2.7 (2.8) | 5.0 (3.3) |
| Lausanne | 61 | 2.4 (1.7) | 2.8 (1.3) |
| Geneva | 47 | 0.9 (1.0) | 3.0 (1.2) |
| Bern | 31 | 2.1 (1.5) | 6.4 (3.3) |


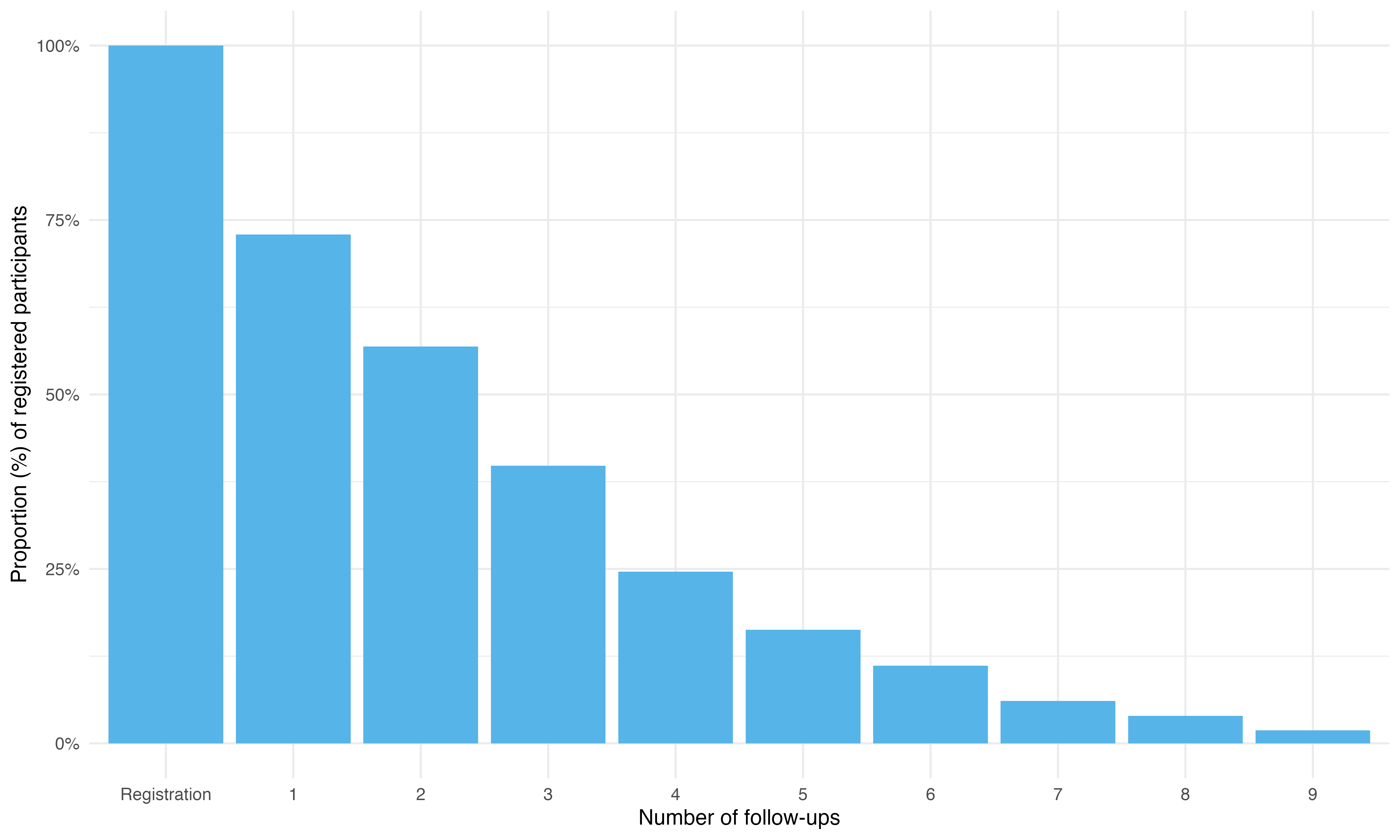


Figure A.1: Proportion of participants by number of follow-ups.

Table A.2: Frequencies of cohort exit types (death or non-death exit) and reasons/causes (total n = 590 exits).

| Exit type | Count (%) | % within exit type  known reasons/causes only |
| --- | --- | --- |
| Non-death* | **460 (78.0)** |  |
| - care by non-cohort physician | 112 (19.0) | 38.1 |
| - patient wish | 76 (12.9) | 25.9 |
| - end of OAT treatment | 45 (7.6) | 15.3 |
| - moved to a foreign country | 23 (3.9) | 7.8 |
| - centre change/closure | 19 (3.2) | 6.5 |
| - prison/deportation | 5 (0.8) | 1.7 |
| - other | 14 (2.4) | 4.8 |
| - unknown reason (lost to follow-up/no contact possible) | 166 (28.1) |  |
| Death | **120 (20.3)** |  |
| - malignancy | 13 (2.2) | 17.8 |
| - overdose of narcotics | 10 (1.7) | 13.7 |
| - liver failure/cirrhosis | 7 (1.2) | 9.6 |
| - COPD (including infection-exacerbated) | 5 (0.8) | 6.8 |
| - pneumonia | 5 (0.8) | 6.8 |
| - sepsis/bacteraemia/endocarditis | 5 (0.8) | 6.8 |
| - suicide | 4 (0.7) | 5.5 |
| - cardiac death | 3 (0.5) | 4.1 |
| - COVID-19 | 2 (0.3) | 2.7 |
| - kidney failure | 2 (0.3) | 2.7 |
| - homicide | 2 (0.3) | 2.7 |
| - other | 15 (2.5) | 20.5 |
| - unknown cause | 47 (8.0) |  |
| Unknown exit type | 10 (1.7) |  |

**An additional 59 participants exited the cohort, but re-entered.*

Table A.3: Distribution of place of death (total n = 120 deaths).

| Place of death | Count (%) | % deaths  with known places |
| --- | --- | --- |
| At home | 49 (40.8) | 46.2 |
| Hospital acute care | 38 (31.7) | 35.8 |
| Institution chronic care | 14 (11.7) | 13.2 |
| Other | 5 (4.2) | 4.7 |
| Unknown place of death | 14 (11.7) |  |

Appendix B: Patient demographics

Table B.1: Patient demographics: 1) at registration, 2) at the last follow-up (including all patients), and 3) latest available data for patients in the cohort at the end of 2024 (i.e., excluding those who died or dropped out of the cohort). Social/job/living variables: during the last 6 months.

| Characteristic |  | *n* | At registration | *n* | At  last follow-up | *n* | Latest data available |
| --- | --- | --- | --- | --- | --- | --- | --- |
| Age |  | 1502 |  | 1502 |  | 912 |  |
| years | median  (IQR) |  | 44.3  (36.1–50.6) |  | 47.9  (38.9–54.7) |  | 50.9  (41.1–57.4) |
| Gender |  | 1501 |  | 1501 |  | 911 |  |
| - male | #  (%) |  | 1137  (75.7%) |  | 1137  (75.7%) |  | 680  (74.5%) |
| - female | #  (%) |  | 364  (24.3%) |  | 364  (24.3%) |  | 231  (25.4%) |
| Ethnicity |  | 1500 |  | 1500 |  | 912 |  |
| - white | #  (%) |  | 1464  (97.6%) |  | 1464  (97.6%) |  | 886  (97.1%) |
| - Asian | #  (%) |  | 18  (1.2%) |  | 18  (1.2%) |  | 15  (1.6%) |
| - Hispano-American | #  (%) |  | 10  (0.7%) |  | 10  (0.7%) |  | 5  (0.5%) |
| - black | #  (%) |  | 5  (0.3%) |  | 5  (0.3%) |  | 5  (0.5%) |
| - other | #  (%) |  | 3  (0.2%) |  | 3  (0.2%) |  | 1  (0.1%) |
| BMI category |  | 1434 |  | 1462 |  | 896 |  |
| - underweight  (<18.5 kg/m^2^) | #  (%) |  | 91  (6.3%) |  | 101  (6.9%) |  | 56  (6.3%) |
| - normal  (18.5–24.9 kg/m^2^) | #  (%) |  | 714  (49.8%) |  | 720  (49.2%) |  | 449  (50.1%) |
| - overweight  (25–29.9 kg/m^2^) | #  (%) |  | 408  (28.5%) |  | 403  (27.6%) |  | 244  (27.2%) |
| - obese  (≥30 kg/m^2^) | #  (%) |  | 221  (15.4%) |  | 238  (16.3%) |  | 147  (16.4%) |
| Unemployed | | 1384 |  | 1435 |  | 909 |  |
| #  (%) | |  | 816  (59.0%) |  | 889  (62.0%) |  | 548  (60.3%) |
| Receiving financial support | | 1310 |  | 1405 |  | 903 |  |
| #  (%) | |  | 1022  (78.0%) |  | 1085  (77.2%) |  | 676  (74.9%) |
| Been imprisoned (for ≥3 months) | | 1394 |  | 1452 |  | 908 |  |
| #  (%) | |  | 78  (5.6%) |  | 66  (4.5%) |  | 38  (4.2%) |
| Been homeless (for ≥3 months) | | 1405 |  | 1455 |  | 908 |  |
| #  (%) | |  | 50  (3.6%) |  | 49  (3.4%) |  | 27  (3.0%) |
| Living with assistance | | 1393 |  | 1438 |  | 907 |  |
| #  (%) | |  | 241  (17.3%) |  | 245  (17.0%) |  | 120  (13.2%) |
| Living in care home | | 239 |  | 357 |  | 194 |  |
| #  (%) | |  | 31  (13.0%) |  | 62  (17.4%) |  | 29  (14.9%) |
| In stable partnership | | 1369 |  | 1428 |  | 900 |  |
| #  (%) | |  | 572  (41.8%) |  | 549  (38.4%) |  | 354  (39.3%) |
| Commercial sex work | | 953 |  | 1087 |  | 705 |  |
| #  (%) | |  | 15  (1.6%) |  | 9  (0.8%) |  | 6  (0.9%) |


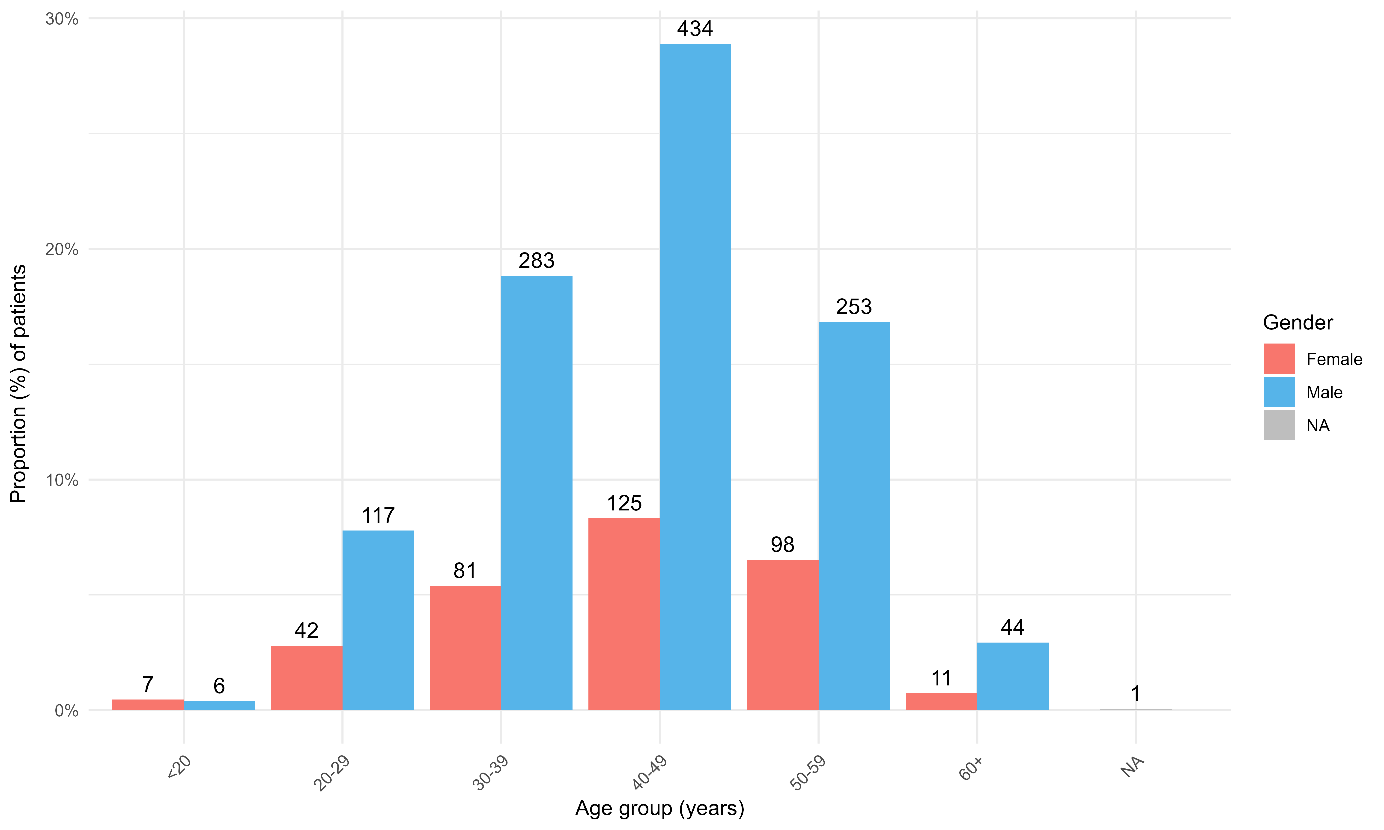


Figure B.1: Age and gender of participants at registration. NA: Not available (missing data).


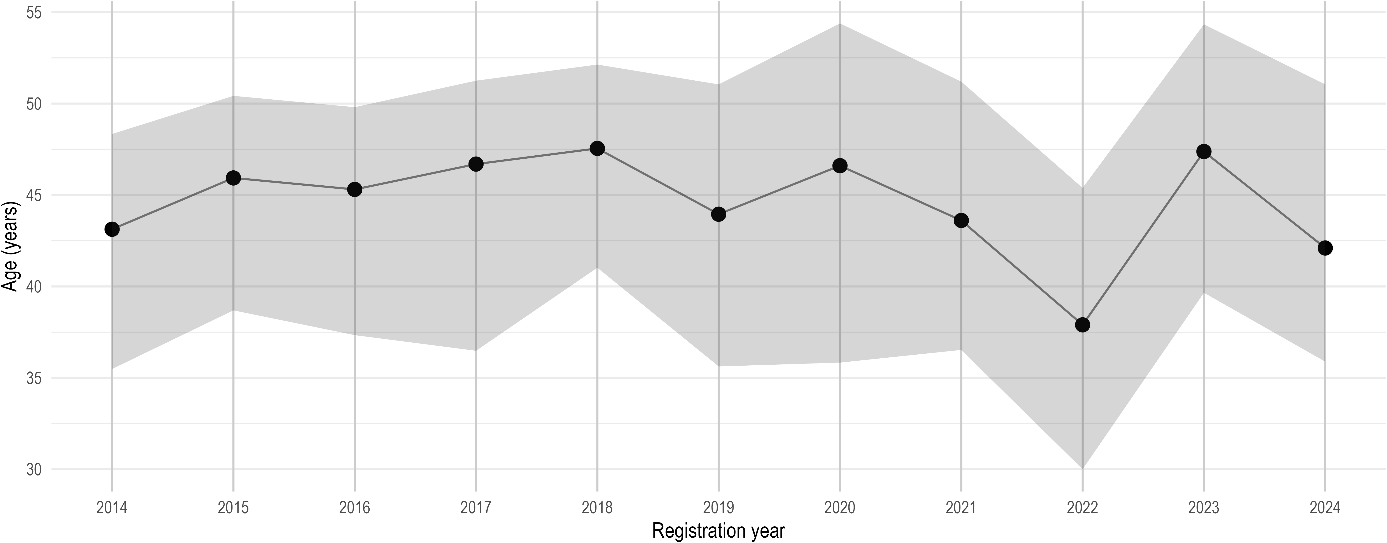


*Figure B.2: Median age at cohort registration (with interquartile range) over time. The median age at registration decreased slightly from 45.8 years in the first five years to 43.0 years in the last five years (while the age of the patients followed in the cohort increased, as shown in the patient demographics table).*

Appendix C: Substance use

Table C.1: Lifetime use of substances and administration routes as reported at registration, with median year of first use and median age at first use.

| Substance/administration route | *n* | Count (%) | Median year of first use | Median age at first use  (years) |
| --- | --- | --- | --- | --- |
| Heroin | 1479 | 1437 (97.2%) | 1993 | 19.4 |
| Cocaine | 1463 | 1351 (92.3%) | 1994 | 19.8 |
| Benzodiazepine | 1448 | 994 (68.6%) | 1995 | 22.4 |
| Cannabis | 1438 | 1291 (89.8%) | 1987 | 15.5 |
| Intravenous drug use | 1464 | 1071 (73.2%) | 1994 | 20.4 |
| Intranasal drug use | 1447 | 1302 (90.0%) | 1993 | 18.7 |
| Nicotine | 1240 | 1204 (97.1%) | 1988 | 14.7 |
| E-cigarette | 357 | 47 (13.2%) | 2020 | 37.5 |


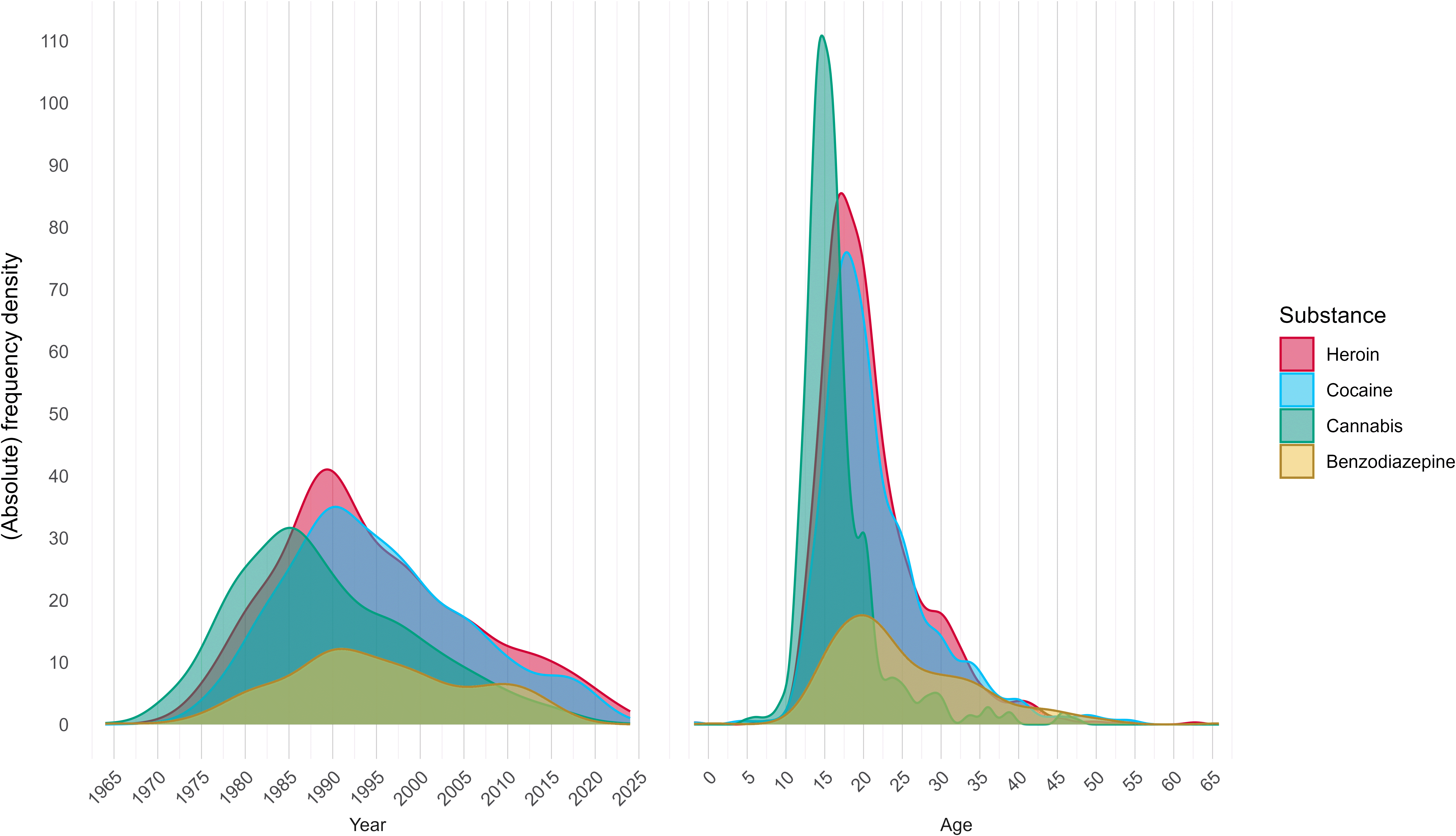


*Figure C.1: Onset of substance use. Year of first use (left) and age (in years) at first use (right), by substance*

*
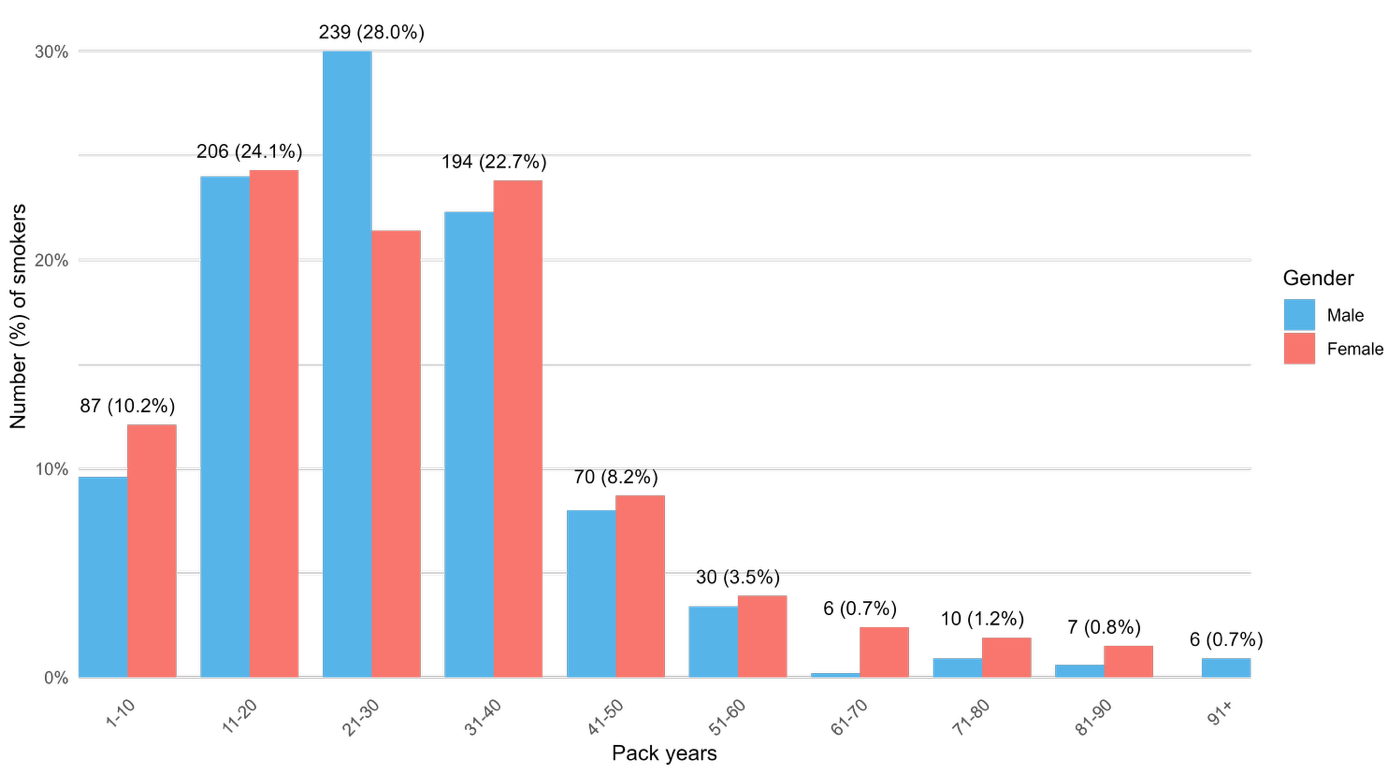
*

Figure C.2: Nicotine use in pack years (pys) at registration, by gender (n = 855 smokers; median = 29 pack years; the numbers and percentages above the bars refer to both sexes combined).


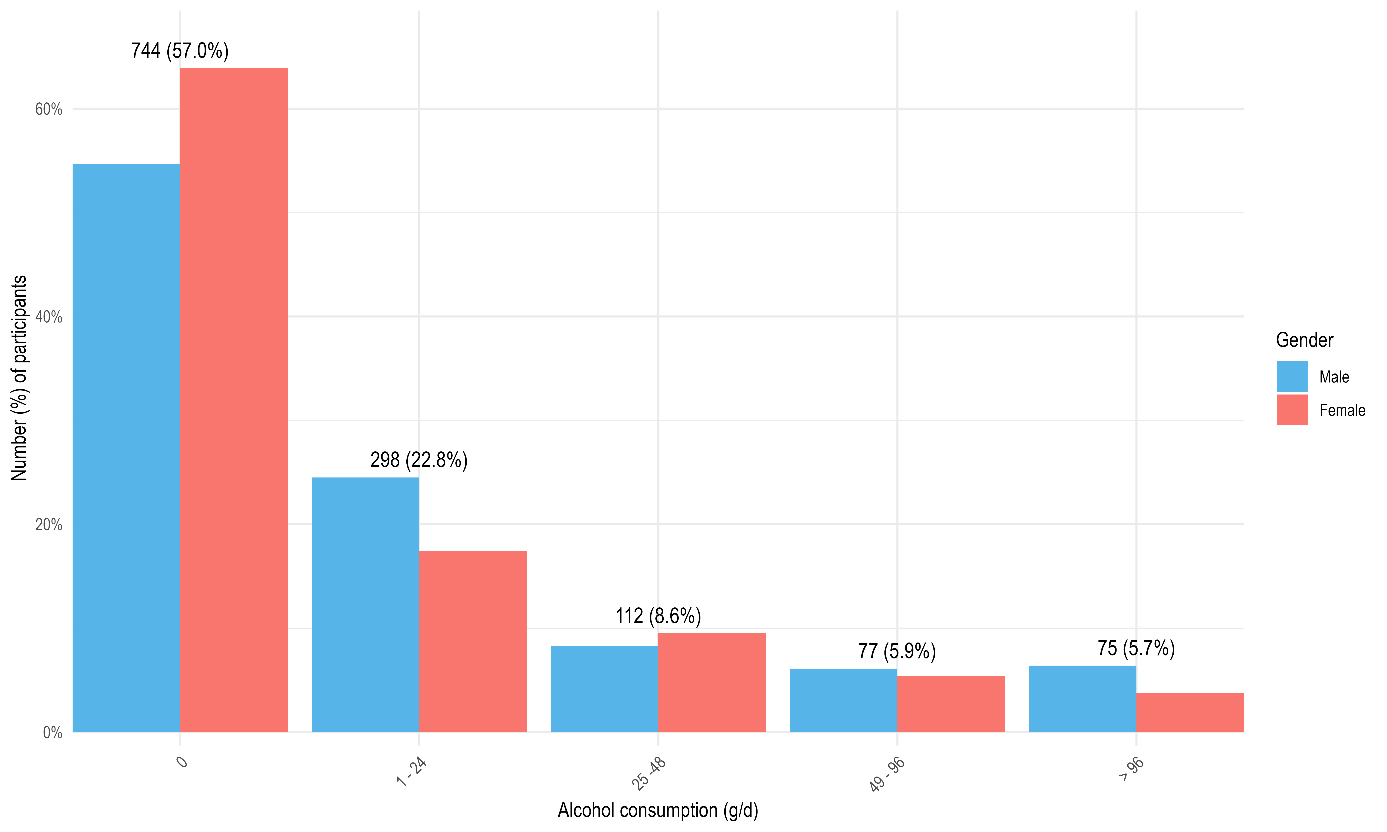


Figure C.3: Alcohol consumption in g/d at registration, by gender (n = 1 306 participants; the numbers and percentages above the bars refer to both sexes combined).

Appendix D: OAT/substitution


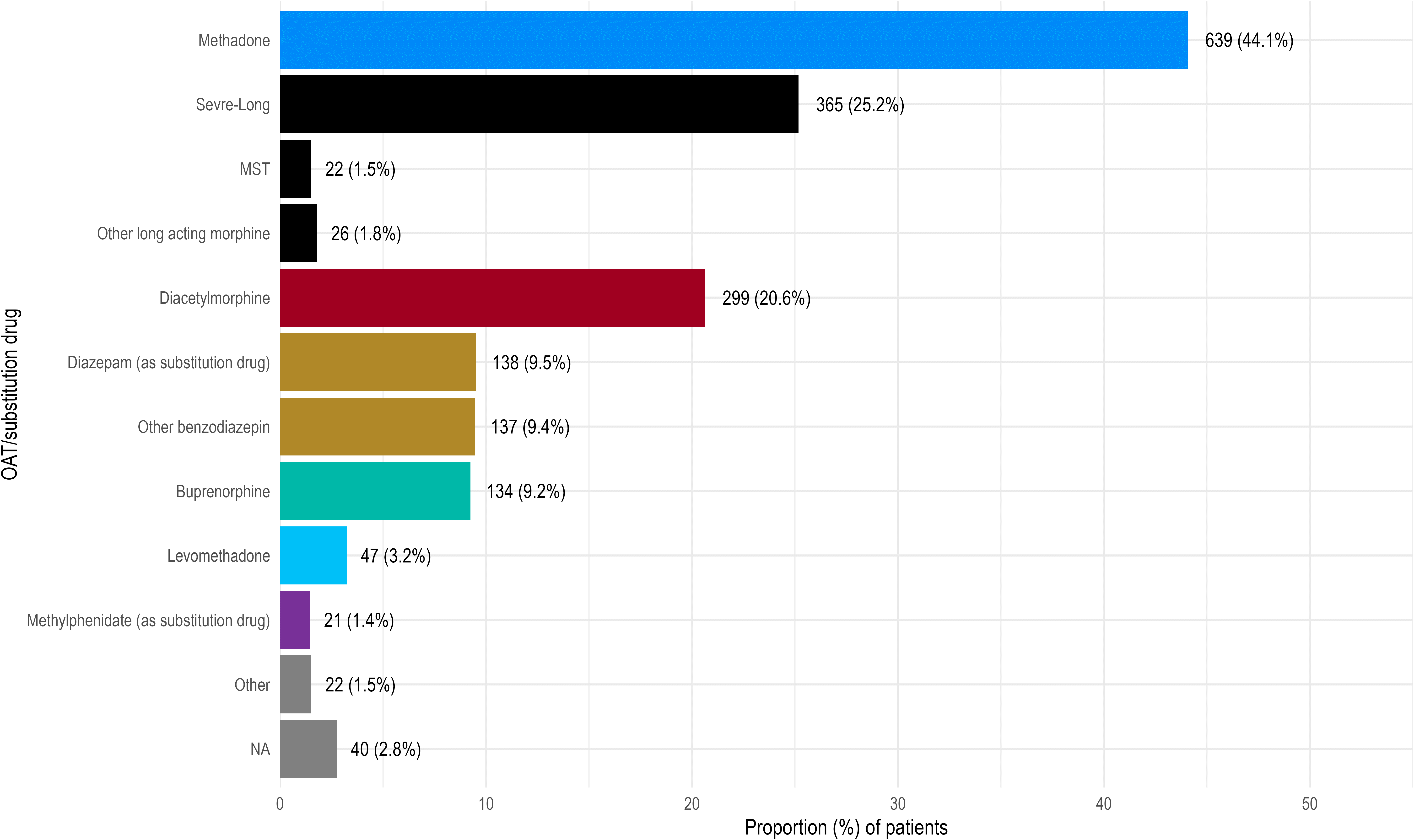


Figure D.1: Number (and percentage) of patients with specific OAT or other substitution medications at the time of cohort registration (adding to > 100% due to some patients having multiple OAT/substitution drugs). NA: Not available (missing data).


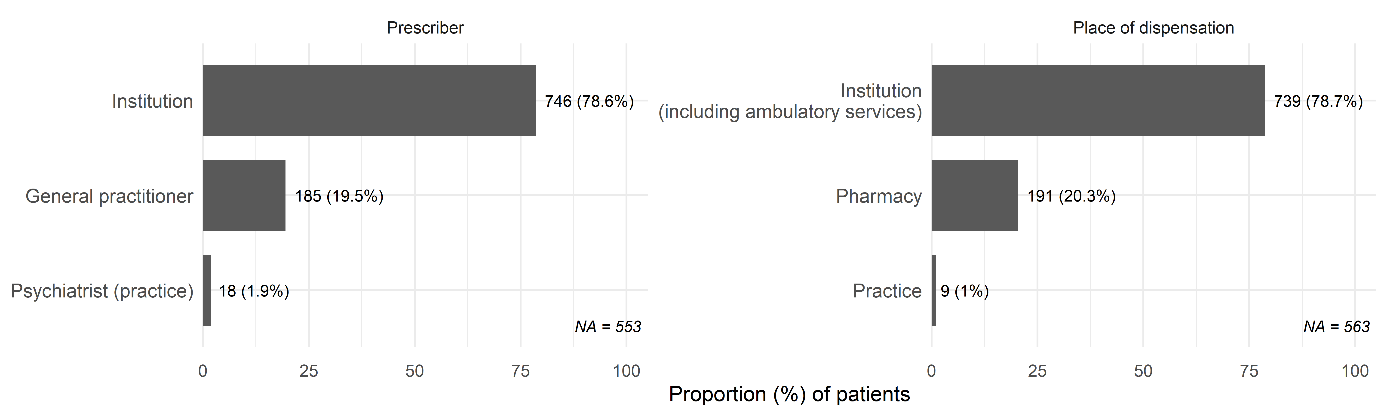


Figure D.2: Prescriber (left) and place of dispensation (right). NA: Not available (high proportion of missing data, as collection of these variables started only in 2021).

Appendix E: Comorbidity

Table E.1: Lifetime prevalence of somatic conditions at registration and at the latest follow-up. Indented entries represent categories (e.g., subtypes or stages), with % still referring to n = no. of participants with available data. For malignancies, the ten, and for cardiovascular diseases, the five most prevalent ICD categories are shown.

| Diagnosis | *n* | Count (%) at registration | *n* | Count (%) at last follow-up |
| --- | --- | --- | --- | --- |
| HCV | 1484 | 839 (56.5%) | 1492 | 880 (59.0%) |
| Major surgery | 1278 | 299 (23.4%) | 1436 | 413 (28.8%) |
| - extremities | | 166 (13.0%) |  |  |
| - abdominal | | 122 (9.5%) |  |  |
| - head |  | 46 (3.6%) |  |  |
| - thoracic | | 38 (3.0%) |  |  |
| Arterial (pre)hypertension | 1285 | 239 (18.6%) | 1437 | 337 (23.5%) |
| - prehypertension (120-139/80-89 mmHg) | | 61 (4.7%) |  |  |
| - stage 1 (140-159/90-99 mmHg) | | 80 (6.2%) |  |  |
| - stage 2 (>155/>99 mmHg) | | 34 (2.6%) |  |  |
| Musculoskeletal disorder | 1337 | 184 (13.8%) | 1457 | 273 (18.7%) |
| Needle abscess | 1262 | 173 (13.7%) | 1434 | 226 (15.8%) |
| Epilepsy | 1486 | 174 (11.7%) | 1497 | 196 (13.1%) |
| - withdrawal or intoxication | | 102 (6.9%) |  |  |
| - genuine (primary) | | 13 (0.9%) |  |  |
| - brain disease | | 8 (0.5%) |  |  |
| COPD | 1283 | 127 (9.9%) | 1436 | 212 (14.8%) |
| - stage A |  | 27 (2.1%) |  |  |
| - stage B |  | 23 (1.8%) |  |  |
| - stage C |  | 7 (0.5%) |  |  |
| - stage D |  | 13 (1.0%) |  |  |
| HIV | 1475 | 135 (9.2%) | 1493 | 136 (9.1%) |
| Malignancies | 1285 | 108 (8.4%) | 1437 | 204 (14.2%) |
| - C50: of the breast | | 27 (2.1%) |  |  |
| - C55: of the uterus, part unspecified | | 12 (0.9%) |  |  |
| - C70: of the meninges | | 12 (0.9%) |  |  |
| - C21: of the anus and anal canal | | 11 (0.9%) |  |  |
| - C22: of the liver and intrahepatic bile ducts | | 11 (0.9%) |  |  |
| - C47: of peripheral nerves and autonomic nervous system | | 11 (0.9%) |  |  |
| - C34: of bronchus and lung | | 10 (0.8%) |  |  |
| - C43: malignant melanoma of skin | | 10 (0.8%) |  |  |
| - C66: of the renal pelvis | | 10 (0.8%) |  |  |
| - C87: of lymphoid, haematopoietic and related tissue | | 10 (0.8%) |  |  |
| Thrombosis | 1287 | 97 (7.5%) | 1438 | 125 (8.7%) |
| - non-septic | | 63 (4.9%) |  |  |
| - septic (with bacteraemia) | | 25 (1.9%) |  |  |
| Hypogonadism | 1080 | 68 (6.3%) | 1340 | 96 (7.2%) |
| Cardiovascular diseases | 1281 | 80 (6.2%) | 1436 | 130 (9.1%) |
| - I25: chronic ischaemic heart disease | | 13 (1.0%) |  |  |
| - I27: other pulmonary heart diseases | | 11 (0.9%) |  |  |
| - I49: other cardiac arrhythmias | | 11 (0.9%) |  |  |
| - I21: acute myocardial infarction | | 9 (0.7%) |  |  |
| - I63: cerebral infarction | | 9 (0.7%) |  |  |
| Osteoporosis | 1269 | 75 (5.9%) | 1433 | 116 (8.1%) |
| Asthma | 1281 | 70 (5.5%) | 1436 | 87 (6.1%) |
| Diabetes mellitus | 1287 | 58 (4.5%) | 1437 | 92 (6.4%) |
| - type I |  | 3 (0.2%) |  |  |
| - type II |  | 47 (3.7%) |  |  |
| - due to specific causes | | 3 (0.2%) |  |  |
| STD | 1277 | 55 (4.3%) | 1435 | 65 (4.5%) |
| - syphilis |  | 21 (1.6%) |  |  |
| - gonorrhoea | | 12 (0.9%) |  |  |
| - chlamydial | | 10 (0.8%) |  |  |
| - other | | 17 (1.3%) |  |  |
| Thyroid dysfunction | 1489 | 59 (4.0%) | 1499 | 72 (4.8%) |
| - hypothyroidism | | 47 (3.2%) |  |  |
| - hyperthyroidism | | 10 (0.7%) |  |  |
| Pancreatitis | 1285 | 32 (2.5%) | 1437 | 53 (3.7%) |
| Endocarditis | 1284 | 25 (1.9%) | 1437 | 39 (2.7%) |
| Peripheral artery disease (PAD) | 1281 | 15 (1.2%) | 1437 | 36 (2.5%) |
| - stage I |  | 3 (0.2%) |  |  |
| - stage II |  | 3 (0.2%) |  |  |
| - stage IV | | 1 (0.1%) |  |  |

Table E.2: Prevalence of psychiatric comorbidities at registration and at the last follow-up. n = number of participants with available data.

| Diagnosis | *n* | Count (%) at registration | *n* | Count (%) at last follow-up |
| --- | --- | --- | --- | --- |
| Affective disorders | 1238 | 431 (34.8%) | 1502 | 598 (39.8%) |
| Personality disorders | 1235 | 287 (23.2%) | 1502 | 351 (23.4%) |
| Anxiety disorders | 1237 | 223 (18.0%) | 1502 | 370 (24.6%) |
| Schizophrenia | 1489 | 261 (17.5%) | 1502 | 299 (19.9%) |
| Suicide attempt(s) | 1468 | 253 (17.2%) | 1502 | 299 (19.9%) |
| Behavioural disorders | 1236 | 192 (15.5%) | 1502 | 419 (27.9%) |
| Attention deficit hyperactivity disorder (ADHD) | 1236 | 142 (11.5%) | 1502 | 186 (12.4%) |


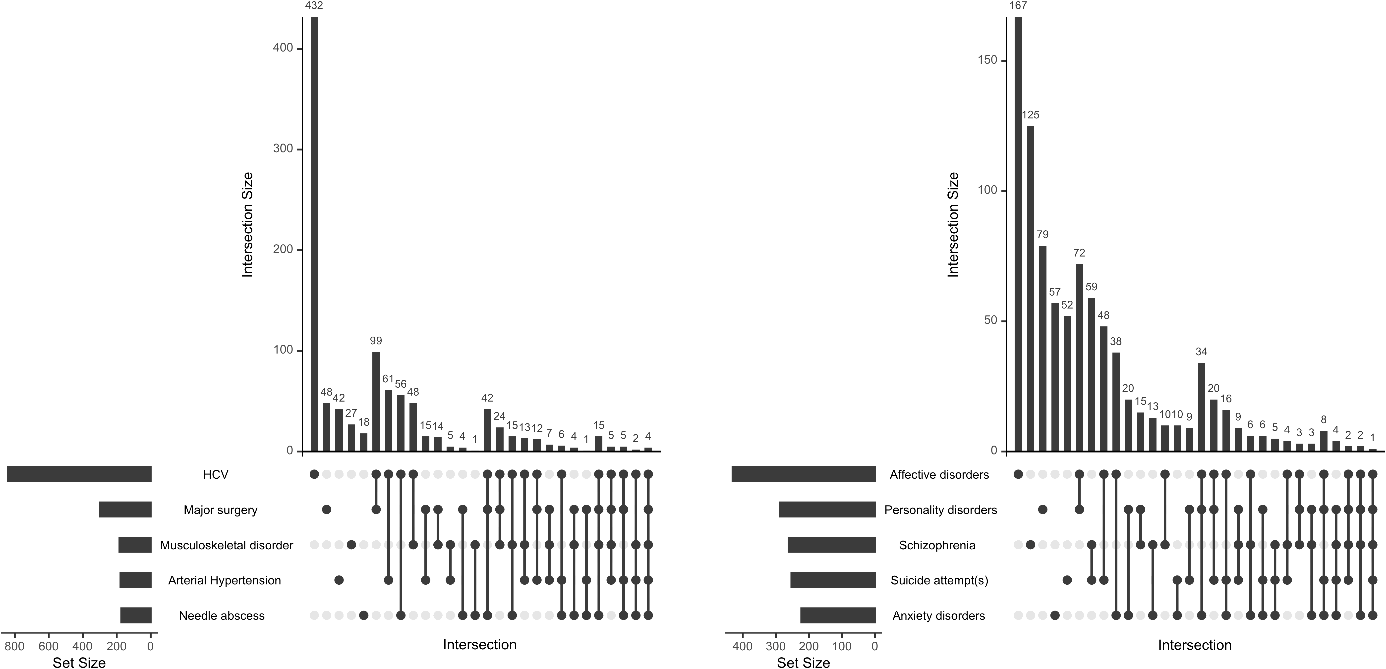


Figure E.1: Co-occurrence of frequent conditions. UpSet plot with patient counts of the five most prevalent somatic (left) and psychiatric (right) conditions at registration (total N = 1 502).

Table E.3: Associations between substance use and somatic/psychiatric diagnoses. For each risk factor (administration route or substance; shown in bold), the table presents the prevalence of diagnoses (indented) in both exposed and non-exposed groups, together with the corresponding (unadjusted) risk ratio (RR) and 95% Wald confidence interval (CI). For ease of reference, only associations with RR ≥ 2.5 are listed.

| Substance/administration route  - diagnosis | Non-exposed group  count (%) | Exposed group  count (%) | RR (95% CI) |
| --- | --- | --- | --- |
| Intravenous drug use | | | |
| - needle abscess | 2 (0.6%) | 169 (18.8%) | 31.5 (7.8-126.1) |
| - HIV | 6 (1.6%) | 123 (11.5%) | 7.2 (3.2-16.2) |
| - HCV | 40 (10.6%) | 780 (72.9%) | 6.9 (5.1-9.3) |
| - peripheral artery disease (PAD) | 1 (0.3%) | 13 (1.4%) | 4.8 (0.6-36.3) |
| - endocarditis | 2 (0.6%) | 23 (2.5%) | 4.2 (1.0-17.7) |
| - sexually transmitted disease (STD) | 5 (1.5%) | 49 (5.4%) | 3.6 (1.4-8.9) |
| - osteoporosis | 7 (2.1%) | 63 (7.0%) | 3.3 (1.5-7.1) |
| - thrombosis | 11 (3.3%) | 85 (9.2%) | 2.8 (1.5-5.2) |
| - pancreatitis | 4 (1.2%) | 28 (3.1%) | 2.6 (0.9-7.2) |
| Intranasal drug use | | | |
| - hypogonadism | 2 (2.0%) | 66 (7.0%) | 3.6 (0.9-14.3) |
| Heroin | | |  |
| - hypogonadism | 0 (0.0%) | 68 (6.6%) | - |
| - osteoporosis | 0 (0.0%) | 71 (5.9%) | - |
| - pancreatitis | 0 (0.0%) | 32 (2.6%) | - |
| - endocarditis | 0 (0.0%) | 25 (2.0%) | - |
| - peripheral artery disease (PAD) | 0 (0.0%) | 15 (1.2%) | - |
| - HIV | 1 (2.5%) | 129 (9.1%) | 3.6 (0.5-25.5) |
| - major surgery | 3 (7.9%) | 292 (23.9%) | 3.0 (1.0-9.0) |
| - needle abscess | 2 (5.3%) | 169 (14.0%) | 2.7 (0.7-10.3) |
| - epilepsy | 2 (4.8%) | 171 (12.0%) | 2.5 (0.6-9.8) |
| Cocaine | | |  |
| - needle abscess | 1 (1.1%) | 170 (14.9%) | 13.9 (2.0-98.1) |
| - asthma | 1 (1.1%) | 68 (5.9%) | 5.5 (0.8-39.4) |
| - epilepsy | 5 (4.6%) | 167 (12.4%) | 2.7 (1.1-6.5) |
| - thrombosis | 3 (3.2%) | 93 (8.0%) | 2.5 (0.8-7.8) |
| Cannabis | | |  |
| - STD | 1 (0.8%) | 54 (4.9%) | 6.2 (0.9-44.5) |
| - epilepsy | 6 (4.1%) | 162 (12.6%) | 3.1 (1.4-6.8) |
| - osteoporosis | 3 (2.4%) | 67 (6.2%) | 2.6 (0.8-8.1) |
| Benzodiazepine | | | |
| - PAD | 2 (0.5%) | 12 (1.4%) | 2.8 (0.6-12.4) |
| - needle abscess | 26 (6.6%) | 142 (17.1%) | 2.6 (1.7-3.8) |
| Nicotine | | |  |
| - COPD | 0 (0.0%) | 124 (10.6%) | - |
| - thyroid dysfunction | 0 (0.0%) | 57 (4.7%) | - |
| - STD | 0 (0.0%) | 54 (4.7%) | - |
| - diabetes mellitus | 0 (0.0%) | 53 (4.5%) | - |
| - pancreatitis | 0 (0.0%) | 32 (2.7%) | - |
| - endocarditis | 0 (0.0%) | 25 (2.1%) | - |
| - PAD | 0 (0.0%) | 15 (1.3%) | - |
| - behavioural disorders | 1 (3.1%) | 180 (15.8%) | 5.1 (0.7-35.0) |
| - epilepsy | 1 (2.9%) | 150 (12.5%) | 4.4 (0.6-30.4) |
| - ADHD | 1 (3.1%) | 132 (11.6%) | 3.7 (0.5-25.7) |
| - major surgery | 3 (9.4%) | 291 (25.0%) | 2.7 (0.9-7.9) |
| E‑cigarette | | | |
| - epilepsy | 24 (7.7%) | 11 (23.4%) | 3.0 (1.6-5.8) |
| - suicide attempt(s) | 38 (12.3%) | 15 (32.6%) | 2.7 (1.6-4.4) |
| - hypogonadism | 13 (4.4%) | 5 (11.4%) | 2.6 (1.0-6.9) |


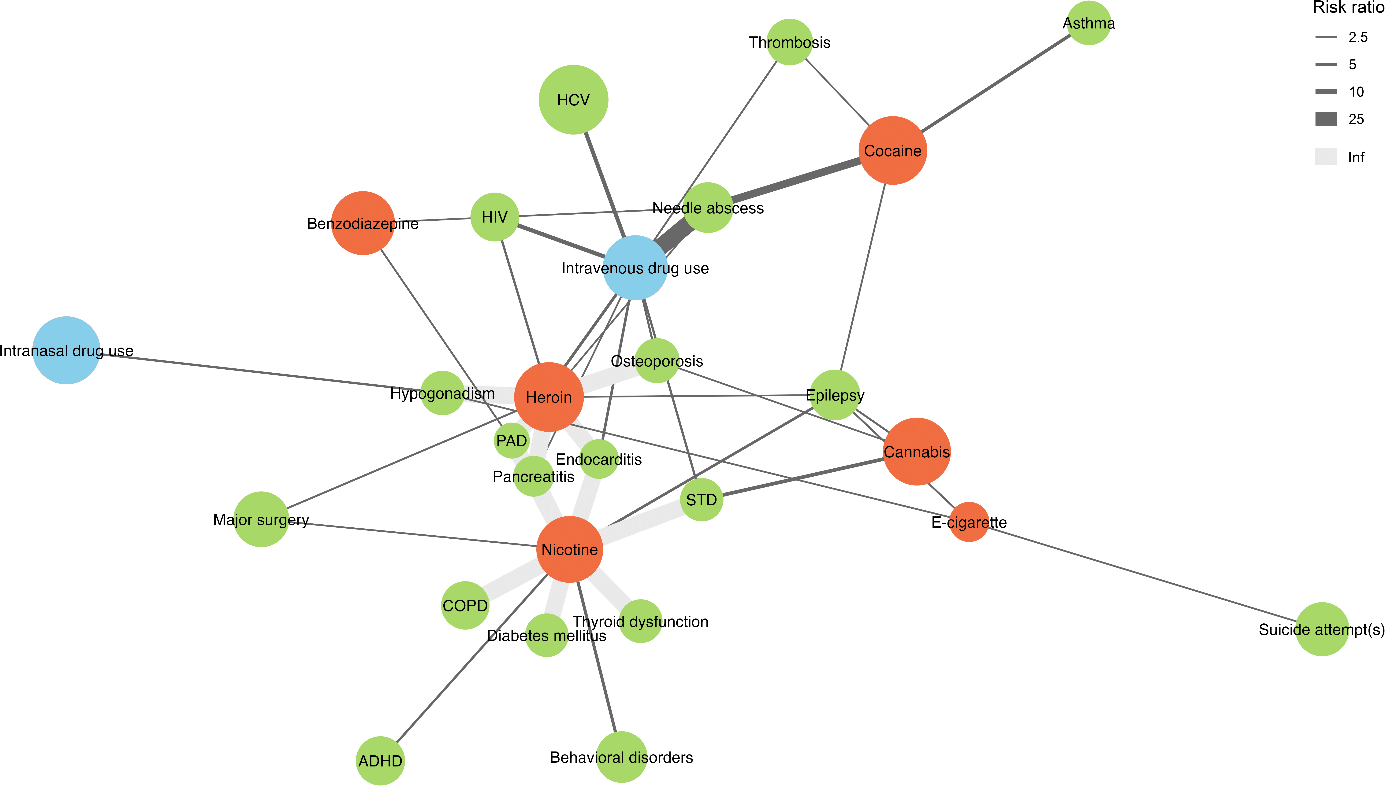


Figure E.2: Network of associations between substances/administration routes and diagnoses. Node size indicates the prevalence of each diagnosis (green nodes), substance (red nodes), or administration route (blue nodes). Edges represent pairwise associations, with edge width proportional to the (unadjusted) risk ratio (RR) of the diagnosis among participants using versus not using the substance or administration route. Light grey edges indicate zero prevalence in the (small) unexposed groups (of non-smokers and non-heroin-users). For clarity and interpretability, only associations with RR ≥ 2.5 are shown.


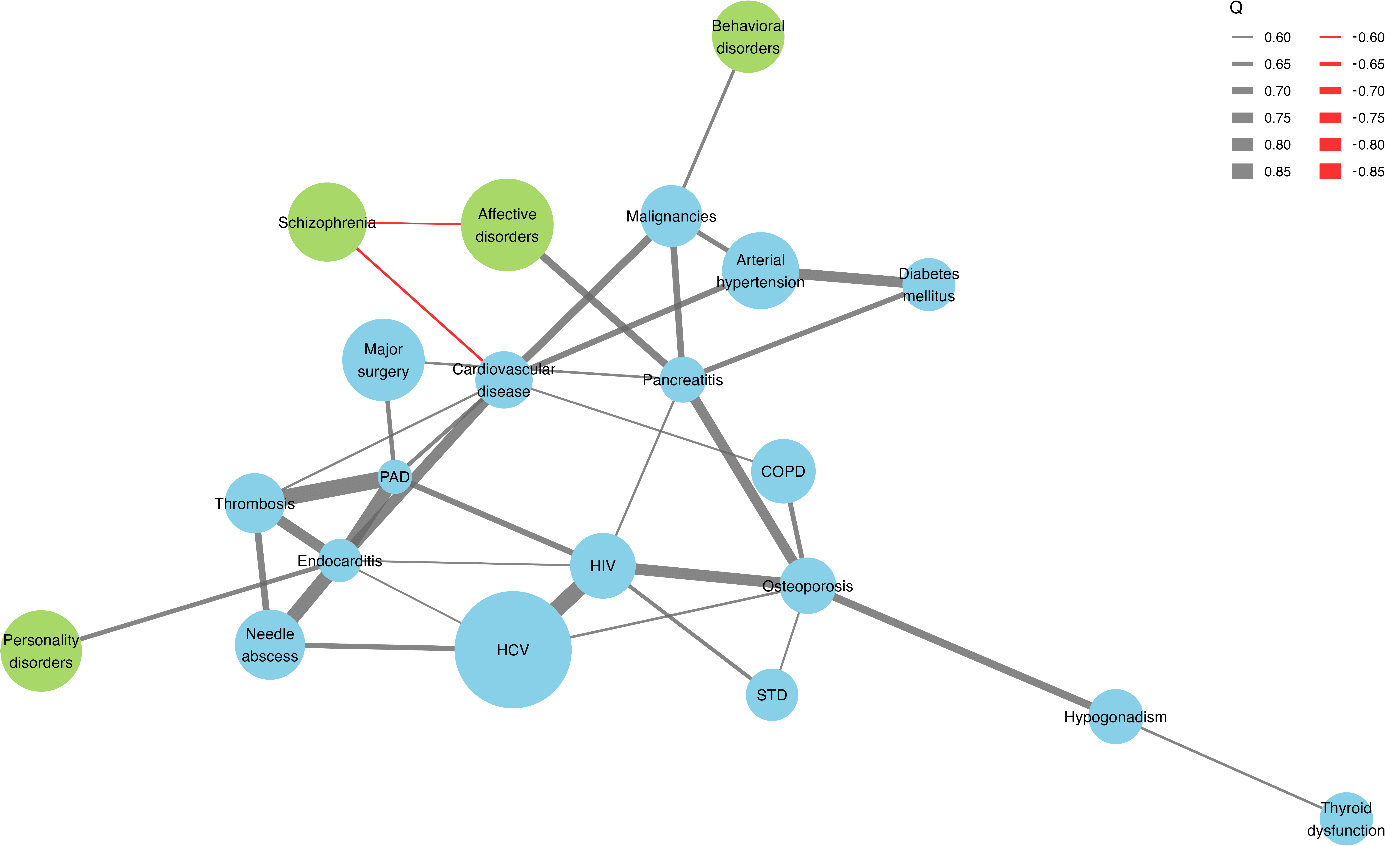


Figure E.3: Network of associations between diagnoses. This visualization emphasizes diagnoses that co-occur either particularly frequently or rarely. Somatic/psychiatric diagnoses are shown as blue/green nodes sized by prevalence. Grey/red edges between nodes indicate strong positive/negative pairwise associations, with edge width reflecting the absolute value of Yule’s Q = (OR–1)/(OR+1) based on unadjusted odds ratios (ORs). Values of Q range between -1 and +1, and a value of zero indicates no association at all. For clarity and interpretability, only associations with |Q| ≥ 0.6 (i.e., OR ≥ 4 or OR ≤ 1/4) are shown.

Appendix F: Medication

Table F.1: Drug classes and subclasses with individual patient counts (prevalence) and prescription counts (proportion of all prescriptions), both for all prescriptions (“ever”) and those active at the end of 2024 (“ongoing”).

|  | Patient count (%) | | | Prescription count (%) | |
| --- | --- | --- | --- | --- | --- |
| Drug class | Ever | | Ongoing | Ever | Ongoing |
| *Total* | *1295 (100.0%)* | | *1171 (100.0%)* | *10655 (100.0%)* | *5448 (100.0%)* |
| Vitamins and minerals | 701 (54.1%) | | 604 (51.6%) | 1756 (16.5%) | 1189 (21.8%) |
| - vitamin D3 | 506 (39.1%) | | 405 (34.6%) | 587 (5.5%) | 421 (7.7%) |
| - folic acid | 144 (11.1%) | | 100 (8.5%) | 157 (1.5%) | 101 (1.9%) |
| - vitamin B12 | 136 (10.5%) | | 108 (9.2%) | 143 (1.3%) | 110 (2.0%) |
| - iron | 130 (10.0%) | | 66 (5.6%) | 145 (1.4%) | 66 (1.2%) |
| - other | 430 (33.2%) | | 339 (28.9%) | 724 (6.8%) | 491 (9.0%) |
| Hepatitis drugs | 582 (44.9%) | | 11 (0.9%) | 1445 (13.6%) | 17 (0.3%) |
| - NS5A inhibitors | 383 (29.6%) | | 6 (0.5%) | 400 (3.8%) | 6 (0.1%) |
| - NS5B inhibitors | 300 (23.2%) | | 4 (0.3%) | 311 (2.9%) | 4 (0.1%) |
| - ribavirin | 216 (16.7%) | | 0 (0.0%) | 269 (2.5%) | 0 (0.0%) |
| - interferons | 184 (14.2%) | | 0 (0.0%) | 230 (2.2%) | 0 (0.0%) |
| - HCV protease inhibitors | 150 (11.6%) | | 2 (0.2%) | 155 (1.5%) | 2 (0.0%) |
| - amantadine | 5 (0.4%) | | 0 (0.0%) | 5 (0.0%) | 0 (0.0%) |
| - emtricitabine | 1 (0.1%) | | 1 (0.1%) | 1 (0.0%) | 1 (0.0%) |
| - entecavir | 1 (0.1%) | | 1 (0.1%) | 1 (0.0%) | 1 (0.0%) |
| - tenofovir | 1 (0.1%) | | 0 (0.0%) | 1 (0.0%) | 0 (0.0%) |
| - other | 62 (4.8%) | | 3 (0.3%) | 72 (0.7%) | 3 (0.1%) |
| Antidepressants | 570 (44.0%) | | 446 (38.1%) | 827 (7.8%) | 536 (9.8%) |
| - SSRI | 320 (24.7%) | | 225 (19.2%) | 386 (3.6%) | 241 (4.4%) |
| - other | 344 (26.6%) | | 254 (21.7%) | 441 (4.1%) | 295 (5.4%) |
| Gastrointestinal drugs | 512 (39.5%) | | 402 (34.3%) | 875 (8.2%) | 530 (9.7%) |
| - proton pump inhibitors | 319 (24.6%) | | 199 (17.0%) | 374 (3.5%) | 199 (3.7%) |
| - laxatives | 223 (17.2%) | | 176 (15.0%) | 300 (2.8%) | 205 (3.8%) |
| - metoclopramide | 22 (1.7%) | | 7 (0.6%) | 27 (0.3%) | 7 (0.1%) |
| - other | 133 (10.3%) | | 99 (8.5%) | 174 (1.6%) | 119 (2.2%) |
| Benzodiazepines | 440 (34.0%) | | 355 (30.3%) | 767 (7.2%) | 487 (8.9%) |
| - diazepam | 131 (10.1%) | | 92 (7.9%) | 139 (1.3%) | 94 (1.7%) |
| - lorazepam | 75 (5.8%) | | 40 (3.4%) | 75 (0.7%) | 40 (0.7%) |
| - zolpidem | 74 (5.7%) | | 51 (4.4%) | 75 (0.7%) | 51 (0.9%) |
| - midazolam | 33 (2.5%) | | 22 (1.9%) | 34 (0.3%) | 22 (0.4%) |
| - clobazam | 10 (0.8%) | | 4 (0.3%) | 10 (0.1%) | 4 (0.1%) |
| - flunitrazepam | 5 (0.4%) | | 5 (0.4%) | 5 (0.0%) | 5 (0.1%) |
| - other | 295 (22.8%) | | 231 (19.7%) | 429 (4.0%) | 271 (5.0%) |
| Antipsychotics | 405 (31.3%) | | 325 (27.8%) | 583 (5.5%) | 399 (7.3%) |
| - quetiapine | 233 (18.0%) | | 175 (14.9%) | 253 (2.4%) | 176 (3.2%) |
| - olanzapine | 59 (4.6%) | | 40 (3.4%) | 63 (0.6%) | 40 (0.7%) |
| - chlorprothixene | 56 (4.3%) | | 38 (3.2%) | 59 (0.6%) | 38 (0.7%) |
| - aripiprazole | 35 (2.7%) | | 24 (2.0%) | 40 (0.4%) | 26 (0.5%) |
| - risperidone | 21 (1.6%) | | 16 (1.4%) | 21 (0.2%) | 16 (0.3%) |
| - clotiapine | 16 (1.2%) | | 11 (0.9%) | 17 (0.2%) | 11 (0.2%) |
| - clozapine | 11 (0.8%) | | 9 (0.8%) | 11 (0.1%) | 9 (0.2%) |
| - other | 104 (8.0%) | | 75 (6.4%) | 119 (1.1%) | 83 (1.5%) |
| Other | 356 (27.5%) | | 256 (21.9%) | 578 (5.4%) | 351 (6.4%) |
| - PDE5 inhibitors | 9 (0.7%) | | 5 (0.4%) | 10 (0.1%) | 5 (0.1%) |
| - blood transfusion | 5 (0.4%) | | 0 (0.0%) | 6 (0.1%) | 0 (0.0%) |
| - other | 349 (26.9%) | | 255 (21.8%) | 562 (5.3%) | 346 (6.4%) |
| Cardiovascular system | 288 (22.2%) | | 247 (21.1%) | 573 (5.4%) | 383 (7.0%) |
| - ACE inhibitors | 127 (9.8%) | | 95 (8.1%) | 149 (1.4%) | 97 (1.8%) |
| - diuretics | 98 (7.6%) | | 70 (6.0%) | 145 (1.4%) | 87 (1.6%) |
| - beta blockers | 74 (5.7%) | | 57 (4.9%) | 88 (0.8%) | 58 (1.1%) |
| - calcium channel blockers | 60 (4.6%) | | 40 (3.4%) | 68 (0.6%) | 40 (0.7%) |
| - angiotensin II receptor blockers | 19 (1.5%) | | 15 (1.3%) | 22 (0.2%) | 15 (0.3%) |
| - other | 74 (5.7%) | | 64 (5.5%) | 101 (0.9%) | 86 (1.6%) |
|  |  | |  |  |  |
| Analgesics/anti-inflammatories | 280 (21.6%) | | 169 (14.4%) | 537 (5.0%) | 235 (4.3%) |
| - NSAIDs | 137 (10.6%) | | 52 (4.4%) | 179 (1.7%) | 54 (1.0%) |
| - paracetamol | 80 (6.2%) | | 44 (3.8%) | 95 (0.9%) | 44 (0.8%) |
| - metamizole | 63 (4.9%) | | 28 (2.4%) | 73 (0.7%) | 28 (0.5%) |
| - acetylsalicylic acid | 14 (1.1%) | | 5 (0.4%) | 14 (0.1%) | 5 (0.1%) |
| - other | 135 (10.4%) | | 86 (7.3%) | 176 (1.7%) | 104 (1.9%) |
| Inhalation drugs | 197 (15.2%) | | 170 (14.5%) | 356 (3.3%) | 256 (4.7%) |
| - short-acting beta-agonist | 78 (6.0%) | | 61 (5.2%) | 86 (0.8%) | 66 (1.2%) |
| - inhaled steroids | 77 (5.9%) | | 56 (4.8%) | 83 (0.8%) | 58 (1.1%) |
| - long-acting beta-agonists | 77 (5.9%) | | 62 (5.3%) | 89 (0.8%) | 62 (1.1%) |
| - other | 77 (5.9%) | | 62 (5.3%) | 98 (0.9%) | 70 (1.3%) |
| Mood stabilizers | 196 (15.1%) | | 175 (14.9%) | 254 (2.4%) | 194 (3.6%) |
| - methylphenidate | 97 (7.5%) | | 82 (7.0%) | 99 (0.9%) | 83 (1.5%) |
| - valproate | 9 (0.7%) | | 5 (0.4%) | 9 (0.1%) | 5 (0.1%) |
| - lithium | 3 (0.2%) | | 3 (0.3%) | 3 (0.0%) | 3 (0.1%) |
| - other | 115 (8.9%) | | 94 (8.0%) | 143 (1.3%) | 103 (1.9%) |
| Metabolism | 154 (11.9%) | | 130 (11.1%) | 275 (2.6%) | 200 (3.7%) |
| - statins | 74 (5.7%) | | 65 (5.6%) | 80 (0.8%) | 67 (1.2%) |
| - metformin | 46 (3.6%) | | 34 (2.9%) | 54 (0.5%) | 35 (0.6%) |
| - other oral antidiabetic | 30 (2.3%) | | 25 (2.1%) | 35 (0.3%) | 28 (0.5%) |
| - insulins | 25 (1.9%) | | 19 (1.6%) | 46 (0.4%) | 29 (0.5%) |
| - other lipid lowering drugs | 17 (1.3%) | | 12 (1.0%) | 17 (0.2%) | 12 (0.2%) |
| - other | 38 (2.9%) | | 27 (2.3%) | 43 (0.4%) | 29 (0.5%) |
| Antiretrovirals | 145 (11.2%) | | 133 (11.4%) | 1099 (10.3%) | 307 (5.6%) |
| - nucleoside/nucleotide analogues | | 110 (8.5%) | 81 (6.9%) | 455 (4.3%) | 141 (2.6%) |
| - HIV protease inhibitors | 90 (6.9%) | | 17 (1.5%) | 272 (2.6%) | 30 (0.6%) |
| - integrase inhibitors | 86 (6.6%) | | 68 (5.8%) | 130 (1.2%) | 68 (1.2%) |
| - non-nucleoside analogues | 47 (3.6%) | | 20 (1.7%) | 67 (0.6%) | 20 (0.4%) |
| - entry inhibitors | 1 (0.1%) | | 0 (0.0%) | 1 (0.0%) | 0 (0.0%) |
| - other | 64 (4.9%) | | 43 (3.7%) | 174 (1.6%) | 48 (0.9%) |
| Antiepileptics | 122 (9.4%) | | 85 (7.3%) | 161 (1.5%) | 101 (1.9%) |
| - levetiracetam | 27 (2.1%) | | 20 (1.7%) | 28 (0.3%) | 20 (0.4%) |
| - valproate | 25 (1.9%) | | 17 (1.5%) | 26 (0.2%) | 17 (0.3%) |
| - lamotrigine | 20 (1.5%) | | 14 (1.2%) | 23 (0.2%) | 14 (0.3%) |
| - phenytoin | 2 (0.2%) | | 0 (0.0%) | 2 (0.0%) | 0 (0.0%) |
| - other | 74 (5.7%) | | 46 (3.9%) | 82 (0.8%) | 50 (0.9%) |
| Anticoagulants | 119 (9.2%) | | 92 (7.9%) | 162 (1.5%) | 99 (1.8%) |
| - direct oral anticoagulants (DOACs) | 48 (3.7%) | | 38 (3.2%) | 57 (0.5%) | 38 (0.7%) |
| - acetylsalicylic acid | 44 (3.4%) | | 36 (3.1%) | 46 (0.4%) | 36 (0.7%) |
| - phenprocoumon | 10 (0.8%) | | 5 (0.4%) | 10 (0.1%) | 5 (0.1%) |
| - parenteral heparin | 9 (0.7%) | | 2 (0.2%) | 10 (0.1%) | 2 (0.0%) |
| - other | 34 (2.6%) | | 18 (1.5%) | 39 (0.4%) | 18 (0.3%) |
| Hormones | 96 (7.4%) | | 75 (6.4%) | 118 (1.1%) | 80 (1.5%) |
| - testosterone | 40 (3.1%) | | 37 (3.2%) | 42 (0.4%) | 37 (0.7%) |
| - thyroid | 28 (2.2%) | | 24 (2.0%) | 30 (0.3%) | 24 (0.4%) |
| - corticosteroids | 8 (0.6%) | | 3 (0.3%) | 8 (0.1%) | 3 (0.1%) |
| - progesterone | 8 (0.6%) | | 3 (0.3%) | 9 (0.1%) | 3 (0.1%) |
| - oestrogens | 6 (0.5%) | | 5 (0.4%) | 6 (0.1%) | 5 (0.1%) |
| - erythropoietin | 3 (0.2%) | | 0 (0.0%) | 3 (0.0%) | 0 (0.0%) |
| - other | 15 (1.2%) | | 8 (0.7%) | 20 (0.2%) | 8 (0.1%) |
| Antibiotics | 85 (6.6%) | | 13 (1.1%) | 162 (1.5%) | 13 (0.2%) |
| - beta-lactam antibiotics | 37 (2.9%) | | 0 (0.0%) | 56 (0.5%) | 0 (0.0%) |
| - quinolones | 11 (0.8%) | | 0 (0.0%) | 12 (0.1%) | 0 (0.0%) |
| - sulfonamides | 10 (0.8%) | | 3 (0.3%) | 13 (0.1%) | 3 (0.1%) |
| - macrolides | 4 (0.3%) | | 0 (0.0%) | 4 (0.0%) | 0 (0.0%) |
| - tuberculostatics | 4 (0.3%) | | 0 (0.0%) | 5 (0.0%) | 0 (0.0%) |
| - other | 51 (3.9%) | | 10 (0.9%) | 72 (0.7%) | 10 (0.2%) |
| Herbals | 66 (5.1%) | | 40 (3.4%) | 74 (0.7%) | 44 (0.8%) |
| Immunomodulators | 29 (2.2%) | | 18 (1.5%) | 36 (0.3%) | 22 (0.4%) |
| Cancer therapy | 7 (0.5%) | | 3 (0.3%) | 10 (0.1%) | 3 (0.1%) |
| Various antiviral drugs | 6 (0.5%) | | 2 (0.2%) | 7 (0.1%) | 2 (0.0%) |


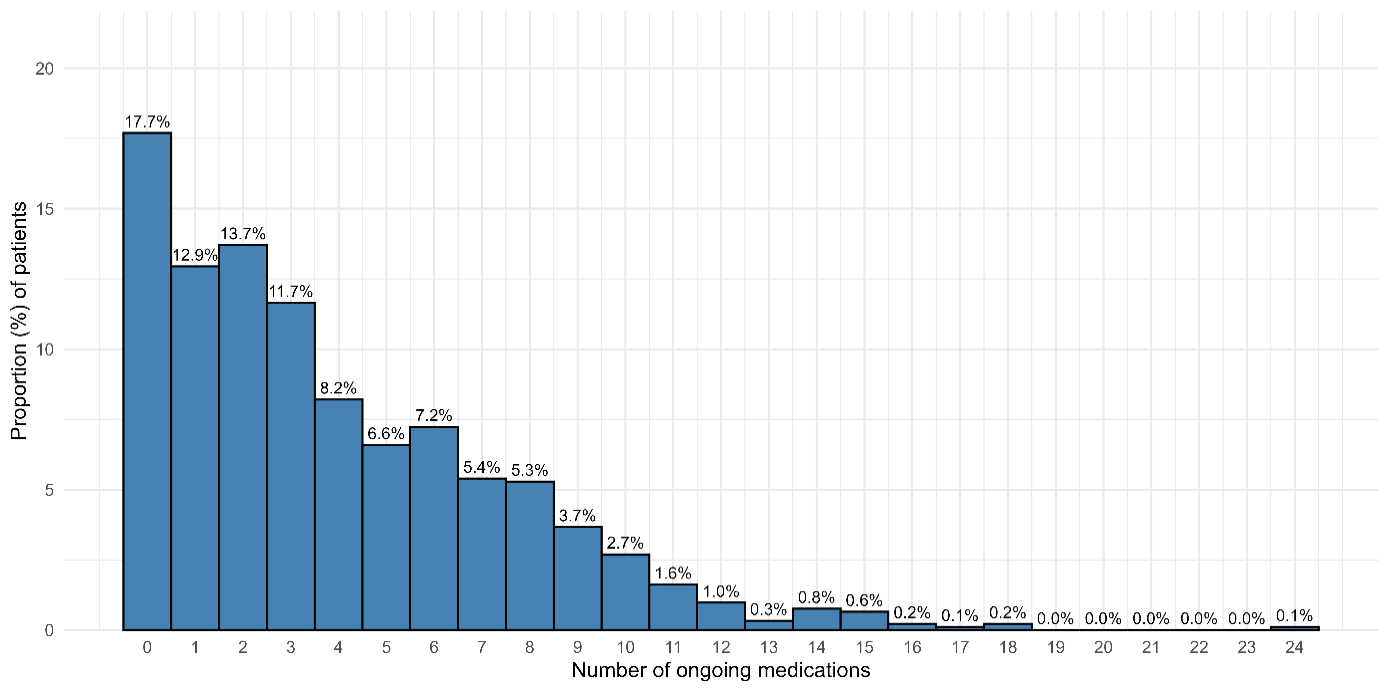


Figure F.1: Number of non-OAT medications per cohort participant (based on last follow-up data) at the end of 2024 (n = 912 participants excluding deaths and dropouts; mean = 3.9).


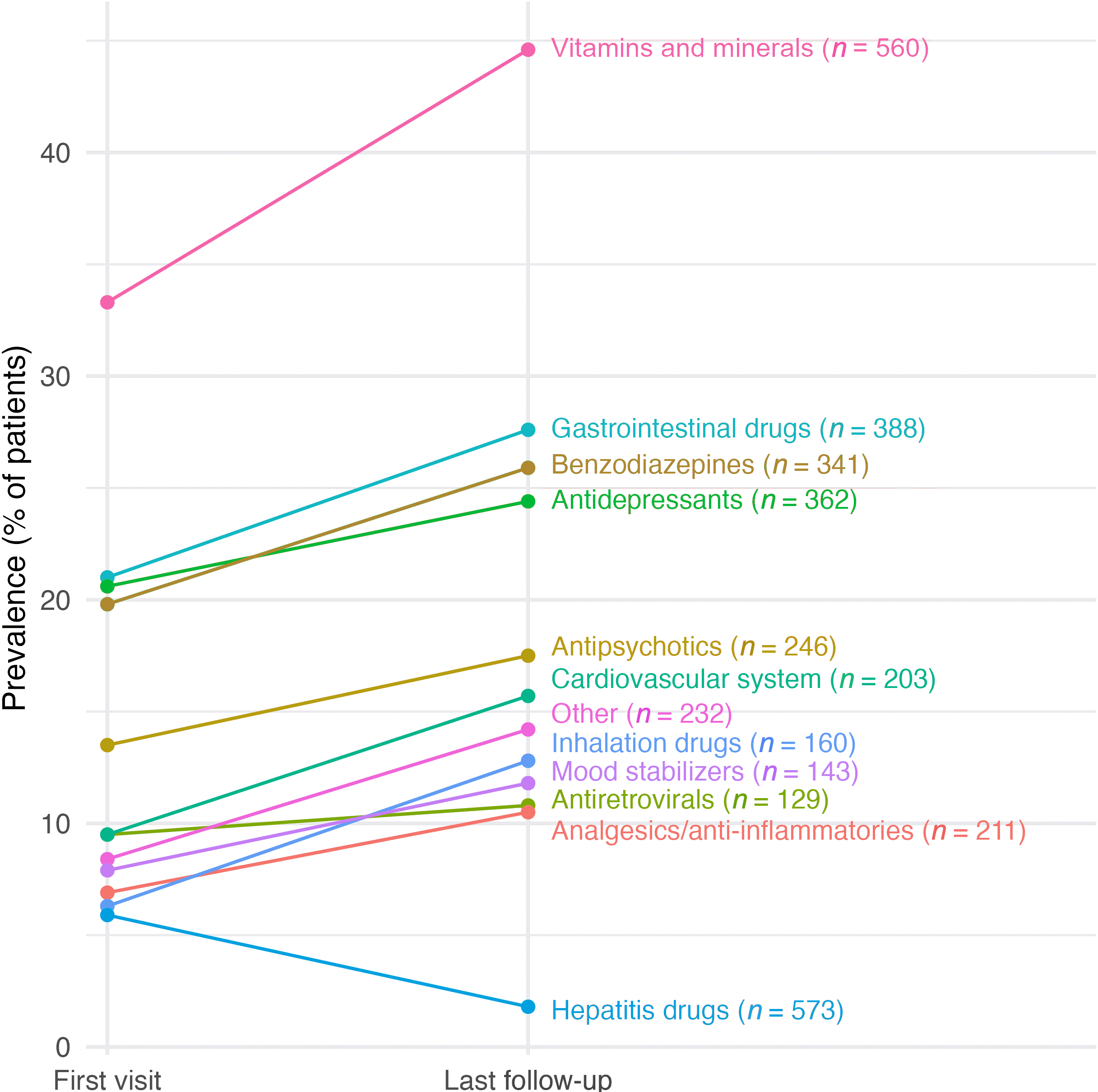


Figure F.2: Prevalence of the twelve most prevalent medication classes at first (= registration) visit and last follow-up. Only n = 1 078 patients with data available from registration and at least one later follow-up visit were considered.

Appendix G: List of variables collected in the SAMMSU cohort

#### Baseline

##### Demographics and eligibility

- Patient ID
- Centre
- Date of visit
- Date of birth
- Age (calculated)
- Gender
- Ethnicity
- Informed consent signed?
- Date of informed consent?

##### Alcohol and drug use (since beginning of drug use)

- Ever intravenous drug use (IVDU)
  - Year of first IVDU
- Ever intranasal drug consumption
  - Year of first intranasal drug consumption
- Ever heroin consumption
  - Year of first heroin consumption
- Ever cocaine consumption
  - Year of first cocaine consumption
- Ever benzodiazepine consumption
- Ever cannabis consumption
  - Year of first cannabis consumption
- Ever nicotine consumption
  - Year of first nicotine consumption
  - Number of pack years
- Ever e-cigarette use
  - Year of first e-cigarette use

##### Somatic comorbidities (before cohort inclusion)

- Ever epilepsy
  - Number of attacks ever
  - Last attack (>/<12 months)
  - Number of attacks last 12 months
  - Etiology (withdrawal or intoxication / brain disease / genuine / unknown)
- Ever thyroid dysfunction
  - specify (hypothyroidism / hyperthyroidism)
- Ever musculoskeletal and connective tissue disorder
  - General ICD-10 code (e.g. M23)
  - Detailed ICD-10 code (e.g. M23.5)
- Ever osteoporosis
  - Osteoporosis / Osteopenia / low bone mass
- Ever thrombosis
  - specify (non-septic / septic)
- Ever endocarditis
  - Number of attacks ever
- Ever cardiovascular disease
  - General ICD-10 code
  - Detailed ICD-10 code
- Ever arterial hypertension
  - specify (Prehypertension / Stage 1 / Stage 2 / not defined)
- Ever peripheral arterial disease
  - specify (Stage I / II / III / IV / not defined)
- Ever COPD
  - Combined GOLD Assessment of COPD category (A / B / C / D / not defined)
- Ever asthma
  - GINA Classification
- Ever diabetes mellitus
  - specify (Type I / Type II / due to specific causes / not defined)
- Ever hypogonadism
- Ever malignancies
  - ICD-10 “C” or “D” code
  - General ICD-10 code
  - Detailed ICD-10 code
- Ever pancreatitis
- Ever STD (except HIV infection)
  - Gonorrhea
  - Syphilis [specify]
  - Chlamydial infection
  - Mycoplasma/Ureaplasma infection
  - Other/undefined STD [specify]
- Ever needle abscess needing surgery
  - Number of surgical interventions for abscess
- Ever major surgery
  - Abdominal surgery
  - Thoracic surgery
  - Head surgery
  - Extremities surgery

##### HIV and HCV

- Anti-HIV positive
  - Ever HIV therapy
  - Year of first HIV therapy
- Anti-HCV positive
  - Ever chronic hepatitis C
  - Ever HCV therapy
  - Year of first HCV therapy

##### Past vaccinations (before cohort inclusion)

- Documentation: Vaccination card available
- Vaccination history (clinical notes) available
- Immunised against Hepatitis A
  - Number of doses received (Hep A)
  - Date of last vaccination (Hep A)
- Immunised against Hepatitis B
  - Number of doses received (Hep B)
  - Date of last vaccination (Hep B)

#### General information

##### Physical examination

- Date of visit
- Age (calculated)
- Height (cm)
- Weight (kg)
- BMI (kg/m²)

##### Alcohol and drug use

- Daily alcohol consumption (g/day)
- Intravenous drug use (IVDU) in the last 12 months
  - Year of first IVDU
  - Frequency of IVDU during the last year
- Intranasal drug consumption in the last 12 months
  - Year of first intranasal drug consumption
  - Frequency of intranasal drug consumption during the last year
- Heroin consumption in the last 12 months
  - Year of first heroin consumption
  - Frequency of heroin consumption during the last year
- Cocaine consumption in the last 12 months
  - Year of first cocaine consumption
  - Frequency of cocaine consumption during the last year
- Benzodiazepine consumption in the last 12 months
  - Frequency of benzodiazepine consumption during the last year
- Cannabis consumption in the last 12 months
  - Year of first cannabis consumption
  - Frequency of cannabis consumption during the last year
- Nicotine consumption in the last 12 months
  - Year of first nicotine consumption
  - Number of pack years
  - Cigarettes per day
- E-cigarette use in the last 12 months
  - Year of first e-cigarette use

##### Drug substitution

- Current participation in drug substitution therapy
- Date of admission to current substitution programme
- Frequency of appearance (days per week)
- Frequency of appearance per day
- Last intake date
- Substitution therapy prescriber
- Place of dispensation
- List of substitution drugs and daily doses

##### Psychiatric comorbidities

- F2 Schizophrenia and other psychotic disorders
- F3 Affective disorders
- F4 Anxiety, adaptation, somatoform disorders
- F5 Behavioural disorders
- F6 Personality disorders
- F90 Attention deficit hyperactive syndrome
- Ever suicide attempt

##### Somatic comorbidities (new since last visit)

- Epilepsy
  - Number of attacks ever
  - Last attack (time frame)
  - Number of attacks in last 12 months
  - Aetiology
- Thyroid dysfunction
  - Type (hypo-/hyperthyroidism)
- Musculoskeletal and connective tissue disorder
  - ICD-10 general code
  - ICD-10 detailed code
- Osteoporosis
  - Osteoporosis / Osteopenia / Low bone mass
- Thrombosis
  - Septic / Non-septic
- Endocarditis
  - Number of attacks ever
- Cardiovascular disease
  - General ICD-10 code
  - Detailed ICD-10 code
- Arterial hypertension
  - Prehypertension / Stage 1 / Stage 2 / not defined
- Peripheral arterial disease
  - Stage I–IV / not defined
- COPD
  - GOLD Assessment category (A–D / not defined)
- Asthma
  - GINA classification
- Diabetes mellitus
  - Type I / Type II / Specific causes / Not defined
- Hypogonadism
- Malignancies
  - ICD-10 type (C/D)
  - General ICD-10 code
  - Detailed ICD-10 code
- Pancreatitis
- STD except HIV
  - Gonorrhoea
  - Syphilis [specify stage]
  - Chlamydial infection
  - Mycoplasma/Ureaplasma infection
  - Other STD [specify]
- Needle abscess needing surgery
  - Number of surgical interventions
- Major surgery
  - Location (abdominal, thoracic, head, extremities)

##### Social network/living situation (last 6 months)

- Stayed in prison (≥3 months)
- Homeless (≥3 months)
- Stable partnership
- Stable partnership with sexual contacts
  - Condom use (always / sometimes / never)
- Occasional sexual contacts
  - Condom use (always / sometimes / never)
- Commercial sex work
  - Condom use (always / sometimes / never)
- Lives alone
- Lives with children <18 years
- Lives with partner
- Lives with parents
- Lives in flat-sharing community
- Lives with assistance
  - Including housing/domestic assistance
  - Including medical assistance
- Living in a care home

##### Job situation

- Unemployed (last 6 months)
- Capacity to work (%)
- Regular job (estimated %, last 6 months)
- Occasional job (estimated hours/week)
- Financial support (last 6 months)
  - Pension
  - Social services
  - Unemployment insurance
  - Invalidity insurance
    - Invalidity score (%)
  - Other financial support (partner, parents, etc.)

#### Medications (including erythropoietin, blood transfusions etc.)

- Medication number
- Drug group (e.g., Cardiovascular system, Antivirals, Antipsychotics, etc.)
- Drug subgroup (e.g., ACE inhibitors, NSAIDs, SSRIs, etc.)
- Specific drug (e.g., Metformin, Valproate, etc.)
- Further specification of drug (optional free-text)
- Start date
- Ongoing (yes/no)
- Stop date (if applicable)
- Reason for stopping treatment

#### **Adverse events**

- Visit date
- Onset of adverse event
- End of adverse event
- Ongoing (yes/no)
- Description of the event
- Intensity (mild/moderate/severe)
- Frequency (single/intermittent/continuous)
- Type of side effect (clinical / laboratory)
- Affected organ system (e.g. psychiatric, neurologic, gastrointestinal)
- Type of event (e.g., anxiety, headache, nausea)
- Grading (1–5, according to CTCAE)
- Type of laboratory side effect
  - entry into corresponding laboratory form (Haematology and Chemistry)
- Relation to medication
  - certain, likely, possible, unlikely, not related
- Medication number (from Medication form)
  - Causality to adverse event
  - Consequences of adverse event
- Action taken: consequences and specific therapy (monitoring, symptomatic treatment, dose reduction, stop drug, none)
  - Outcome (resolved without sequel/resolved with sequel/unknown/other

#### Vaccinations

- Immunised against hepatitis (A / B)
  - Hepatitis vaccine name
  - Date of hepatitis vaccination
- COVID-19 vaccine name (Moderna / Biontech/Pfizer / AstraZeneca / other [specify])
  - Date of COVID-19 vaccination
- Comments (free text)

#### HIV and HCV care

- Anti-HIV test result (negative/positive)
  - Anti-HIV test material (venous / capillary / saliva)
  - Date of anti-HIV test
- Anti-HCV test result (negative/positive)
  - Anti-HCV test material (venous / capillary / saliva)
  - Date of anti-HCV test
- Is the patient Anti-HIV positive?
  - SHCS number known?
  - SHCS number
  - First anamnestic positive HIV test (date)
  - First documented positive HIV test (date)
  - Currently on HIV therapy?
  - Who mainly provides HIV care? (nobody / HIV specialist associated with substitution programme / HIV specialist in hospital / HIV specialist in private practice / family doctor)
- Is the patient Anti-HCV positive?
  - SCCS number known?
  - SCCS number
  - First anamnestic positive HCV test (date)
  - First documented positive HCV test (date)
  - Ever chronic hepatitis C?
  - Currently chronic hepatitis C?
  - Ever HCV reinfection?
  - Number of prior HCV therapies (excluding current)
  - Currently under HCV therapy?
  - Who provides HCV care? (nobody / HCV specialist associated with substitution programme / HCV specialist in hospital / HCV specialist in private practice / family doctor)
- If the physician would currently advise against HCV-therapy, please specify the main reasons:
  - desire to have children?
  - pregnancy or lactation?
  - uncontrolled substance use?
  - uncontrolled alcohol use?
  - uncontrolled psychiatric disorder?
  - uncontrolled somatic disease?
  - unstable life situation?
  - non-compliance with appointments?
  - insufficient patient motivation?
  - other? [specify]
- Comments (free text)

#### HCV therapies

- Start date of the HCV therapy
- Stop date of the HCV therapy
- Ongoing (yes/no)
- Medication number (from medication form)
- Was/is the treatment interferon-based?
- RVR during HCV therapy (undetectable HCV RNA after 4 weeks)
- Partial EVR during HCV therapy (≥2 log drop in HCV RNA after 12 weeks)
- Complete EVR during HCV therapy (undetectable HCV RNA after 12 weeks)
- Outcome:
  - SVR (sustained virological response, i.e. HCV-RNA-negative ≥12 weeks after end)
  - Relapse
  - Breakthrough (reoccurrence of HCV-RNA while still under treatment)
  - Partial response
  - Nonresponse
  - Failure, not defined
- Preterm stop (yes/no)
- Reason for preterm stop:
  - toxicity/complication related to HCV treatment
  - medical complication not related to HCV treatment
  - patient’s wish
  - unknown reasons
- Adherence problems during treatment (yes/no)
- HCV therapy prescriber
  - Infectious disease specialist
  - Gastroenterologist
  - Other prescribers per FOPH list
  - General practitioner (single or group practice)
  - Psychiatrist (practice or institution)
  - None of the above
- Setting of HCV therapy
  - Referral to a specialist
  - Treatment on-site
- Type of on-site treatment
  - Institution (outpatient)
  - Institution (inpatient)
  - General practitioner
  - Psychiatrist (practice)
  - Pharmacy
  - Prison
- Comments (free-text)

#### HCV reinfections

- Date of HCV reinfection diagnosis
- Level of certainty of reinfection (definite/probable)
- HCV reinfection diagnosis after (spontaneous clearance/Successful IFN-based treatment/Successful IFN-free treatment)
- Most likely reason for reinfection (Unsafe intravenous drug use/Unsafe intranasal drug use/Unsafe anal intercourse/Other)
- Outcome of HCV reinfection (Spontaneous clearance/Chronic infection/Unknown)
- Comments (free-text)

#### Diagnostic procedures

- Date of fibroscan
  - Liver stiffness (kPa)
  - IQR (interquartile range) of liver stiffness
  - Total number of fibroscan measurements
  - Number of valid fibroscan measurements
  - Success rate of fibroscan (%)
- Date of liver biopsy
  - Activity grade (Metavir)
  - Fibrosis grade (Metavir)
- Date of sonography
  - Compatible with cirrhosis (yes/no)
  - Focal lesions (yes/no)
  - Ascites (yes/no)
  - Splenomegaly (>11×7×4 cm) (yes/no)
  - Width of the portal vein (mm)
  - Grade of steatosis (I / II / III / not specified)
- Date of gastroscopy
  - Oesophageal varices (yes/no)
- Date of ECG
  - QTc time (ms)
- Date of FRAX score
  - FRAX score for major osteoporotic fracture (%)
  - FRAX score for hip fracture (%)
- Comments (free text)

#### Serology

- Anti-HAV-total result (negative/positive)
  - Date of anti-HAV-total test
- Anti-HAV-IgG result (negative/positive)
  - Date of anti-HAV-IgG test
- Anti-HBs-IgG qualitative result (negative/positive)
  - Date of anti-HBs-IgG qualitative test
- Anti-HBs-IgG quantitative result (IU/l)
  - Date of anti-HBs-IgG quantitative test
- Anti-HBc result (negative/positive)
  - Date of anti-HBc test
- HBsAg result (negative/positive)
  - Date of HBsAg test
- HBeAg result (negative/positive)
  - Date of HBeAg test
- Anti-HBe result (negative/positive)
  - Date of anti-HBe test
- Anti-HDV result (negative/positive)
  - Date of anti-HDV test
- Comments (free text)

#### Hematology and chemistry

- Date of blood count and clotting test
  - Haemoglobin (g/l)
  - Leucocytes / white blood cells (G/l)
  - ANC (absolute neutrophile count) (G/l)
  - Platelets (G/l)
  - INR
- Date of liver value test
  - ASAT (U/l)
  - ALAT (U/l)
  - GGT (U/l)
  - Alkaline phosphatase (U/l)
  - Bilirubin (µmol/l)
  - AFP (µg/l)
- Date of lipid and glucose test
  - Fasting (yes/no)
  - Total cholesterol (mmol/l)
  - HDL cholesterol (mmol/l)
  - Triglycerides (mmol/l)
  - LDL cholesterol (mmol/l)
  - Glucose (mmol/l)
  - HbA1c (NGSP) (%)
- Date of additional general chemistry test
  - Creatinine (µmol/l)
  - Uric acid (µmol/l)
  - Amylase (U/l)
  - Albumin (g/l)
- Date of vitamin and mineral test
  - Vitamin B12 (pmol/l)
  - Vitamin D (25-OH-cholecalciferol) (nmol/l)
  - Ferritin (µg/l)
  - Soluble transferrin receptor (mg/l)
- Comments (free text)

#### Virology and Immunology

- Date of HIV RNA test
  - HIV RNA below level of measurement (yes/no)
  - HIV RNA unit (copies/ml or log copies/ml)
  - HIV RNA result (copies/ml)
  - HIV RNA result (log copies/ml)
- Date of HCV antigen test
  - HCV antigen below level of measurement (yes/no)
  - HCV antigen result (fmol/l)
- Date of HCV RNA test
  - HCV RNA below level of measurement (yes/no)
  - HCV RNA unit (U/ml or log U/ml)
  - HCV RNA result (U/ml)
  - HCV RNA result (log U/ml)
  - HCV RNA material (venous / capillary [direct] / capillary [dried spot])
- Date of HCV genotype test
  - HCV genotype number (1–7)
  - HCV genotype letter (a–d, multiple subtypes, subtype not defined)
- Date of HBV DNA test
  - HBV DNA below level of measurement (yes/no)
  - HBV DNA unit (U/ml or log U/ml)
  - HBV DNA result (U/ml)
  - HBV DNA result (log U/ml)
- Date of HDV RNA test
  - HDV RNA below level of measurement (yes/no)
  - HDV RNA unit (U/ml or log U/ml)
  - HDV RNA result (U/ml)
  - HDV RNA result (log U/ml)
- Date of IL28B polymorphism test
  - IL28B polymorphism result (CC / CT / TT)
- Date of CD4 count (absolute)
  - CD4 count (cells/μl)
- Date of CD4 count (%)
  - CD4 count (%)
- Comments (free text)

#### Centre change

- SAMMSU institution up to now (Aarau / Basel / Bern / Geneva / Lausanne / Lugano / St. Gallen / Zürich / other)
- Other institution (free text)
- New SAMMSU institution
- Other new institution (free text)
- Desired date of centre change
- Comments (free text)

#### Study stop

- Stop date
- Type of stop (drop-out / death)
- Reason for drop-out (moved to a foreign country / end of substitution treatment / patient wish / care by non-cohort physician / other)
- Date of death
- Age at death (calculated)
- Reason for death (HCV / HIV / suicide / overdose of narcotics / accident / homicide / bacteraemia or endocarditis / unknown / other [specify])
- Place of death (hospital acute care / at home / institution chronic care / other [specify])
- Last date the patient was known to be alive
- Age at last known alive date (calculated)
- Re-entry of the patient after drop-out?
  - Re-entry date
  - Re-consent date (needed if drop-out was due to patient wish)
- Patient status (Freeze / Active [= unfreeze])
- Comments (free text)
